# Trajectory-based global sensitivity analysis in multiscale models

**DOI:** 10.1038/s41598-024-64331-x

**Published:** 2024-06-17

**Authors:** Valentina Bazyleva, Victoria M. Garibay, Debraj Roy

**Affiliations:** Faculty of Science, Informatics Institute, University of Amsterdam, Science Park 904, Amsterdam, 1098 XH North Holland The Netherlands

**Keywords:** Complex systems analysis, Global sensitivity analysis, Agent-based models, Sobol’ indices, Grassmannian diffusion maps, Sparse polynomial chaos expansion, Mathematics and computing, Computational science

## Abstract

This research introduces a novel global sensitivity analysis (GSA) framework for agent-based models (ABMs) that explicitly handles their distinctive features, such as multi-level structure and temporal dynamics. The framework uses Grassmannian diffusion maps to reduce output data dimensionality and sparse polynomial chaos expansion (PCE) to compute sensitivity indices for stochastic input parameters. To demonstrate the versatility of the proposed GSA method, we applied it to a non-linear system dynamics model and epidemiological and economic ABMs, depicting different dynamics. Unlike traditional GSA approaches, the proposed method enables a more general estimation of parametric sensitivities spanning from the micro level (individual agents) to the macro level (entire population). The new framework encourages the use of manifold-based techniques in uncertainty quantification, enhances understanding of complex spatio-temporal processes, and equips ABM practitioners with robust tools for detailed model analysis. This empowers them to make more informed decisions when developing, fine-tuning, and verifying models, thereby advancing the field and improving routine practice for GSA in ABMs.

## Introduction

Agent-based models (ABMs) are applied in many disciplines and valued for their capacity to incorporate individual-level heterogeneity, interactions, and adaptability, which makes them a unique tool for studying the actions of individual elements within a system. ABMs are intentionally designed such that agents adapt their actions to information from each other and their environment, enabling researchers to study the emergence of patterns and adaptations at the systemic level. In their basest form, ABMs produce output at micro (agent) and macro (model) levels. If the behavior of clusters or communities is also of interest, a meso level is introduced. ABMs can represent complex adaptive systems with a comprehensive and fluid description of how these systems change over time. The resulting richness in temporal dynamics of ABM output and its multi-levelness make it inherently difficult to fully accommodate with traditional global sensitivity analysis (GSA) methods.

With the insights GSA provides on the contribution of parameters to model uncertainty, models and experiments can be more efficiently developed. The importance of conducting GSA is exemplified in one uncertainty quantification study of a controversial epidemiological model which was instrumental in policy decisions for COVID-19 containment^1^; analysts discovered that only 19 of the 940 input parameters had a significant impact on the model results. In one step towards improving GSA for ABMs, researchers proposed a method of time-varying GSA to capture changes in parametric sensitivities over the course of a simulation rather than relying on, what has become customary, analysis of the final time step^2^. However, even this analysis type can be challenging to interpret when model responses exhibit non-linear behavior (e.g., oscillations, tipping points, etc.), as the resulting sensitivity indices oscillate or appear chaotic. Time-varying GSA fails to generalize the history of the model process or capture its underlying temporal dynamics, providing no clear information on what parameters shift and reshape response curves^3^. Moreover, to date, no established method specifically addresses the multi-levelness of ABMs by offering parameter sensitivity estimation at agent and community levels. Spatial variance-based GSA offers a potential direction, yet adapting it to non-spatially explicit ABMs remains challenging. We refer the reader to the in-depth discussion of the challenges of GSA in ABMs and their consequences in the SI.

For ABMs, the state of the system at a given time is determined by the attributes of individual agents which evolve throughout the simulation, similar to how positions and velocities of particles in a molecular dynamics simulation change over time. Therefore, a comparison can be made between the inner processes of an ABM and a molecular dynamics propagator. Like the propagator, the Koopman operator also offers a framework to analyze system dynamics, enabling the identification of patterns and behaviors without the need to track each state variable’s trajectory explicitly. While the Koopman operator transforms nonlinear dynamics into a linear framework, dynamic mode decomposition (DMD) and Grassmannian diffusion maps (GDMaps) provide methodologies for extracting and analyzing these dynamics in high-dimensional data. Using GDMaps on the high-dimensional output of an ABM, we achieve a low-dimensional approximation of the high-dimensional structures and underlying metastable dynamics. Projecting data onto the Grassmann manifold enables the computation of a low-rank approximation of the high-dimensional system dynamics. Interpolating DMD modes is akin to interpolating on the Grassmann manifold, hence estimating sensitivity indices with data in the latent space is similar to performing GSA on the propagator, allowing systematic generalization of rich temporal dynamics in ABMs. For further details on the Koopman operator, DMD, and their application in this context, more information is available in the SI.

**Figure 1:**
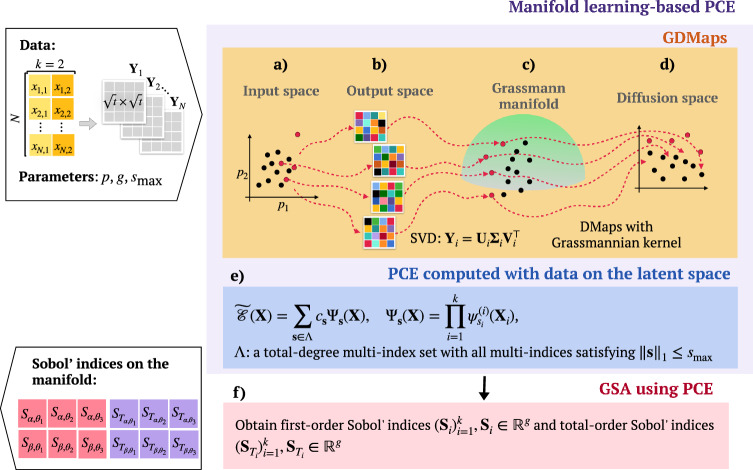
A schematic illustrating the entire pipeline of GSA using PCE on a low-dimensional manifold discovered with GDMaps for two uncertain parameters. The workflow is demonstrated through several key steps: (**a**) generation of parameter samples using Sobol sequences within the input space, (**b**) execution of the computational model to produce high-dimensional output trajectories, represented as matrices, (**c**) projection of these high-dimensional outputs onto the Grassmann manifold to ascertain subspace structures, followed by application of the diffusion maps technique to uncover the nonlinear geometry inherent in a diffusion manifold, (**d**) Utilization of diffusion coordinates for crafting a lower-dimensional embedding that effectively captures crucial aspects of the model output trajectories, (**e**) building a PCE surrogate model with the projected data as output, and (**f**) calculation of first- and total-order sensitivity indices using the coefficients from the PCE surrogate model, which links input parameters to diffusion coordinates, illustrating the model’s sensitivity to each parameter and their interactions. Interpretation of the first-order sensitivity indices as measures of uncertainty reduction in the model output when a parameter is fixed, whereas total-order indices quantify the residual uncertainty attributable to the parameter and its interactions with others.

Our research proposes a novel GSA approach tailored to the distinct features of ABMs, addressing their multi-level nature and rich temporal dynamics. It leverages manifold learning to capture ABM dynamics effectively at different levels of model output, showcasing the potential of these methods in uncertainty quantification. Inspired by the analogy between ABMs and molecular dynamics propagators, we employ GDMaps to identify low-dimensional data representation. We then apply sparse polynomial chaos expansion (PCE) to map uncertain input parameters to the coordinates on the diffusion manifold, obtaining variance-based sensitivity indices on the manifold from PCE coefficients without additional computational effort (see Fig. 1). By expanding the analytical toolbox available to ABM researchers, this method encourages the adoption of sensitivity analysis as a routine practice in the development and exploration of ABMs, aligning with the ABMs research community’s code of conduct^4^.

## Results

Here, we present the results of the proposed GSA framework which uses GDMaps and PCE for complex high-dimensional systems. The first-order sensitivity index ($S_i$) of a parameter measures the reduction in uncertainty of the model output trajectory if that parameter is fixed. Total-order sensitivity indices ($S_{T_i}$) measure the uncertainty remaining in the model output trajectory due to the parameter and its interactions with other parameters. Rather than limiting the results content to one example or the relevance of the proposed method to a narrow audience, three different models were selected to emphasize the generalizability of the framework. We have performed a GSA using the proposed framework on a classical dynamical system (Lotka–Volterra^5^), and two ABMs: DeepABM-COVID^6^ and a poverty trap formation ABM^7^. The first two models are used to demonstrate how the framework is applied to study trajectory-based sensitivities. The last model produces outputs at the individual (micro) level, and facilitates analysis at the community (meso) level, and the population (macro) level, thus we investigate Sobol’ indices resulting from applying the framework at three distinct levels. The models are highlighted for the unique focal characteristics their outputs exhibit, namely oscillation, multiple dependent variables, and multi-levelness, as specified in the following subsections.

### Parametric sensitivities for oscillating trajectories

**Figure 2:**
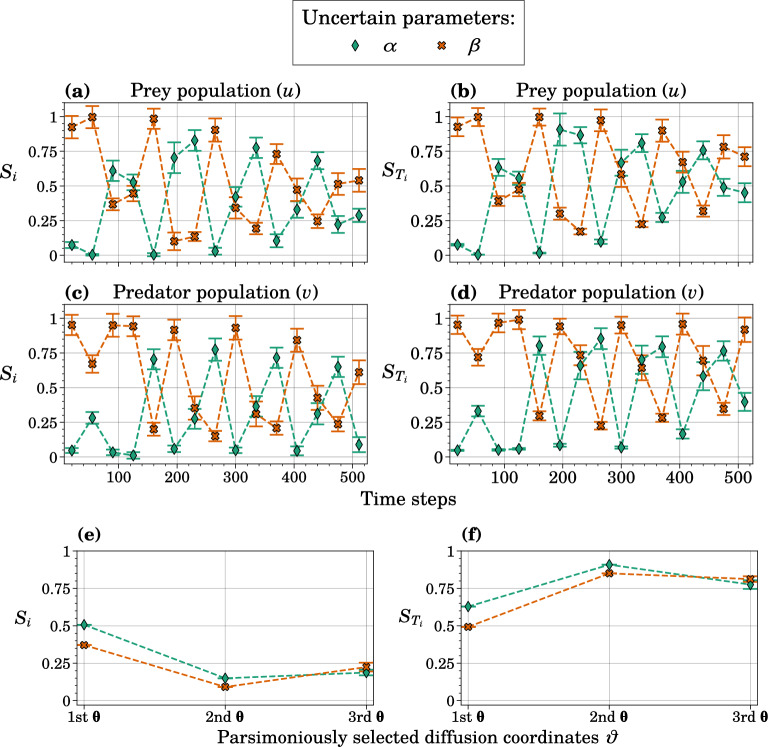
GSA results for the Lotka–Volterra model with two uncertain parameters. (**a**–**d**) First- and total-order sensitivity indices, $S_i$ and $S_{T_i}$, respectively, for $\alpha$ and $\beta$ concerning two model output measures: the number of prey per time step, *u*, and the number of predators per time step, *v*. This assessment is carried out using traditional Sobol’ index calculation methods, and error bars represent 95%-bootstrap confidence intervals. (**e**,**f**) Sensitivity indices for $\alpha$ and $\beta$ with respect to the same model output data using the GSA framework with GDMaps PCE. The error bars represent the variance obtained from 50 resamples. For GDMaps PCE, a Grassmannian dimension of $p=10$ and a maximum polynomial degree of $s_{\max }=6$ were applied.

For the classic Lotka–Volterra system, first- and total-order sensitivity indices and their corresponding 95% bootstrap confidence intervals were calculated at alternating time steps for the driving parameters $\alpha$ and $\beta$ (Fig. 2a–d). The oscillation of the model outputs based on the tested parameter ranges was reflected in the sensitivity indices (see SI, Supplementary Fig. S2). The similarity between first- and total-order index results indicated that main effects, as opposed to parameter interactions, contributed the most to model output variance.

The GSA framework was used in conjunction with GDMaps PCE to compute the mean and variance of first-and total-order sensitivity indices based on 50 resampled input matrices (Fig. 2e,f). We focused on the results pertaining to the parsimonious selection of diffusion coordinates. A non-parsimonious selection is a common convention for retaining diffusion coordinates based on the magnitude of their corresponding eigenvalues, assuming that the leading eigendirection serves as a representation of the underlying manifold. Parsimonious representation overcomes the issue that certain eigenvectors are essentially higher-order harmonics of earlier eigenvectors and do not introduce new directions within the dataset, suggesting the presence of “repeated eigendirections”, which can complicate the identification of the true dimensionality of the underlying manifold^8^. The 2D plots featured in SI, Supplementary Fig. S3a–c demonstrate convergence of non-trivial, parsimoniously selected diffusion coordinates to $\vartheta = \{\varvec{\theta }_1, \varvec{\theta }_2, \varvec{\theta }_5\}$, dependent on the resampled solution. The PCE coefficients based on each of these retained coordinates were used to derive first- and total-order sensitivity indices. While $\alpha$ sensitivity is slightly higher for the first two diffusion coordinates, the values for $\alpha$ follow closely those of $\beta$ in the results for both main and interaction effects. Total-order results for the second and third diffusion coordinates reveal substantial interaction effects.

The sensitivity indices computed on the manifold using three selected diffusion coordinates indicate parameter influence on the system’s shortest time-scale dynamics, associated with lower eigenvalues. The eigenvalues provide a measure of the significance of these dynamics, with larger eigenvalues corresponding to slower and more important changes in trajectories, while smaller eigenvalues signify shorter time scales and more noise. The first diffusion coordinate for Lotka–Volterra is interpretable as the Hamiltonian of the system (conserved over time). In other words, it is the direction representing the change in the trajectory of the model when the parameters ($\alpha$ and $\beta$) are perturbed. As shown in Fig. 2e,f, both parameters have similar sensitivity indices. Given that the governing equations are additive in parameters—this is expected and provides validation of our method.

Although it is not suitable for comparison with Fig. 2a–d because of the distinctiveness of the data representations, the presented framework emphasizes, arguably more effectively, the varying impact of parameters on model output trajectory. We show in Fig. 2a–d that the (first- and total-order) sensitivity indices vary across time. Since the standard practice is to use the value of a model output at a given point in time (snapshot) as the QoI, the resulting indices would depend on the choice of snapshot, as shown. Therefore, different modelers with different snapshots of the same model may arrive at inconsistent conclusions regarding the order and importance of parameters. Further, the order (importance) of parameters varies with the output chosen (prey vs predator). Since sensitivity indices are often used to remove unimportant parameters from the model or prioritize data collection, it is paramount that these indices are “temporally robust” and are independent of the choice of snapshot. In contrast, our trajectory-based method can identify diffusion coordinates for long-timescale evolution of the system and provide consistent and robust first- and total-order Sobol’ indices (Fig.  2e,f).

### Sensitivity indices for multiple dependent outputs

**Figure 3:**
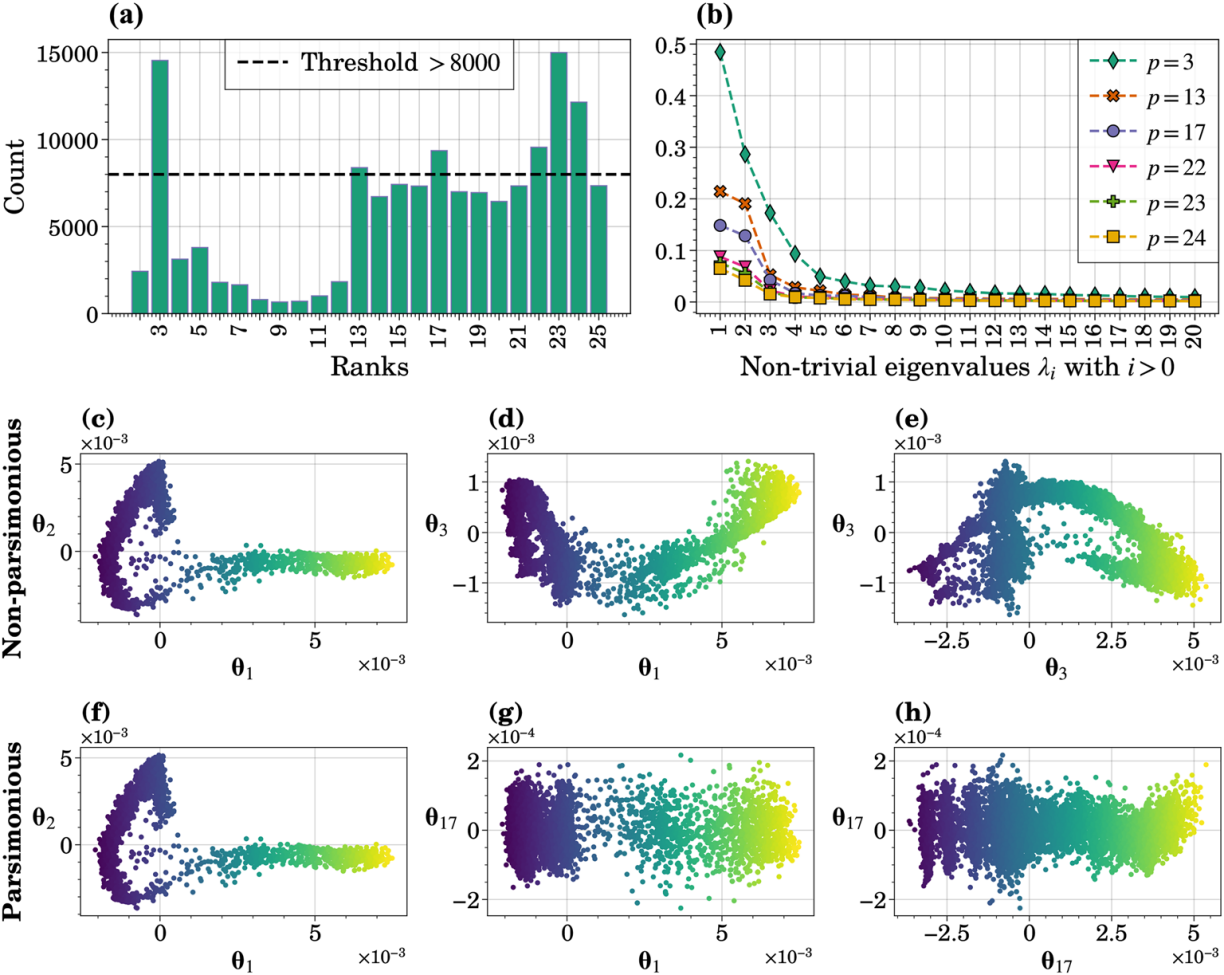
Preliminary analysis of data generated from running DeepABM-COVID with five uncertain parameters. (**a**) The distribution of rank occurrences across the entire dataset, combining data from 20 runs, with a threshold to determine the Grassmannian dimension, *p*. (**b**) Scree-plot displaying eigenvalues derived from GDMaps applied to the DeepABM-COVID model output matrix, $\mathcal{Y} \in \mathbb {R}^{7168 \times 900}$ for run 3. Six different Grassmannian dimensions, *p*, were considered. (**c**–**h**) 2D plots illustrating the behavior of three diffusion coordinates from GDMaps when applied to the DeepABM-COVID model output for run 16 and Grassmannian dimension of $p=13$. In figures (**f**–**h**), the diffusion coordinates are selected through a parsimonious approach, resulting in $\vartheta = \{\varvec{\theta }_1, \varvec{\theta }_2,\varvec{\theta }_{17}\}$.

The process for data generation, details on uncertain parameters, and an overview of the GSA framework can be found in Methods (“Applications” section). Six dimensions were explored with GDMaps: $p \in \{3, 13, 17, 22, 23, 24\}$, corresponding to the most frequently occurring ranks in the complete dataset of 20 runs, illustrated in Fig. 3a together with the selection threshold. Figure 3b features the non-trivial eigenvalues for the selected dimensions. Larger Grassmannian dimensions, *p*, correspond to lower eigenvalues, particularly for $i=1$ and $i=2$, owing to a more detailed data representation on the Grassmannian manifold.

We narrowed down our choice of dimensions for sensitivity index estimation to $p \in \{3, 13, 23\}$; $p=13$ is close to $p=17$, and $p=23$ is close to $p\in \{22,24\}$. We explored both parsimonious and non-parsimonious implementations to retain three diffusion coordinates at the GDMaps step, presenting the results as 2D plots in Fig. 3c–h. It is important to note that the primary difference in these implementations lies in the selection of the third diffusion coordinates. Parsimonious selection corresponds to shorter timescale dynamics, as evidenced by the *y*-axis scale. Additional 2D plots for other Grassmannian dimensions can be found in the SI, Supplementary Figs. S6 (non-parsimonious) and S7 (parsimonious). When using the parsimonious representation for larger *p* values, diffusion coordinates with lower corresponding eigenvalues are selected more frequently, consistent with the behavior observed in Fig. 3b, where higher dimensions displaying lower decay rates are associated with smaller initial eigenvalues.

The 2D plots displaying diffusion coordinates against each other offer a helpful tool for visualizing reduced data. However, interpreting them can be challenging due to the abstract nature of reduced spaces in high dimensions. The presence of coherent and smooth patterns within these plots signifies that GDMaps has effectively captured meaningful structural dynamics within the data. Conversely, a scatter plot resembling a two-dimensional Gaussian distribution indicates that the associated diffusion coordinates represent a strong noise component identified by GDMaps. To check for noise, one can examine the distributions of diffusion coordinates, as illustrated in SI, Supplementary Figs. S8 and S9, where multi-modal distributions correspond to higher eigenvalues, while unimodal and Gaussian-like distributions are associated with lower eigenvalues.

**Figure 4:**
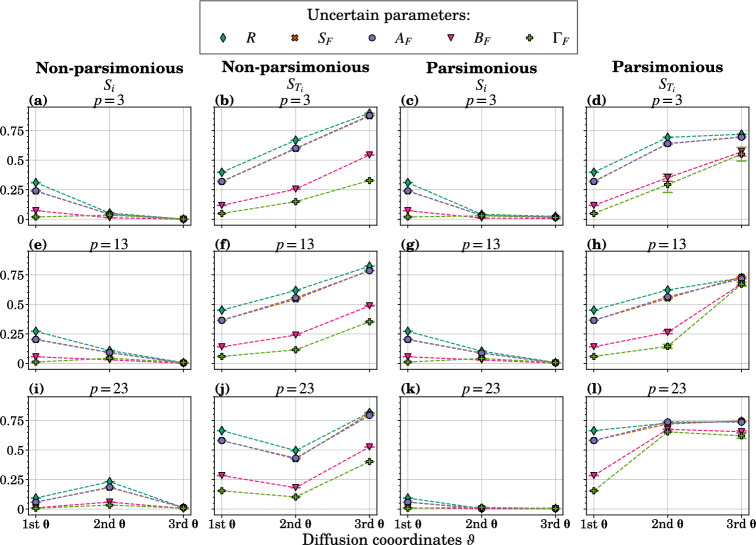
GSA results for DeepABM-COVID. First- ($S_i$) and total-order ($S_{T_i}$) Sobol’ indices for five uncertain input parameters (see Table 2) and five combined model output measures. These indices were computed on the manifold using the GSA framework with GDMaps PCE, and error bars indicate the variance observed over 20 runs.

Figure 4 displays the 20-run average of first- and total-order sensitivity indices obtained from GSA using GDMaps PCE for two methods of retaining diffusion coordinates with a maximum polynomial degree of $s_{\max }=15$. Additional results pertaining to varying maximum polynomial degrees (i.e., $s_{\max } \in {3, 6, 9, 12}$) and estimates of generalization errors are accessible in Supplementary Figs. S10–S13.

We observe that $S_i$ and $S_{T_i}$ are similar across representations for $p=3$ and $p=13$ (Fig. 4a–h). This similarity is attributed to the behavior of GDMaps with lower manifold dimensions, corresponding to larger initial eigenvalues and leading to the parsimonious selection of diffusion coordinates, which capture longer timescale dynamics more frequently. However, the most apparent dissimilarity between the two methods for selecting diffusion coordinates is evident for $p=23$. Larger manifold dimensions yield a more detailed representation of data on the Grassmanian, which could be linked to the potential influence of individual parameters on variance in model output, as can be seen for the second diffusion coordinates in Fig. 4i. Generally, this influence is relatively modest in contrast to the impact of interactions—a finding that concurs with the results obtained with traditional approaches for computing Sobol’ indices (SI, Supplementary Fig. S5). Notably, between the two methods, the order of parameters and their relative importance is comparable. However, Supplementary Fig. S5 illustrates the challenges inherent in current methodologies, wherein sensitivity indices are computed across five distinct outputs derived from a conventional SIR-type DeepABM-COVID model. Our analysis reveals that these sensitivity indices lack temporal robustness, much like observations in the Lotka–Volterra system. Additionally, the order of the indices varies across different outputs, rendering the identification of crucial parameters challenging, if not impossible—a problem which will be further aggravated by increasing dimension of the output space. In contrast, our novel GSA approach allowed us to aggregate the five model output trajectories in three diffusion coordinates, identify the most influential parameters, and capture the strong interaction effects in the second and third diffusion coordinates.

### Sensitivity analysis for multi-level trajectories

**Figure 5:**
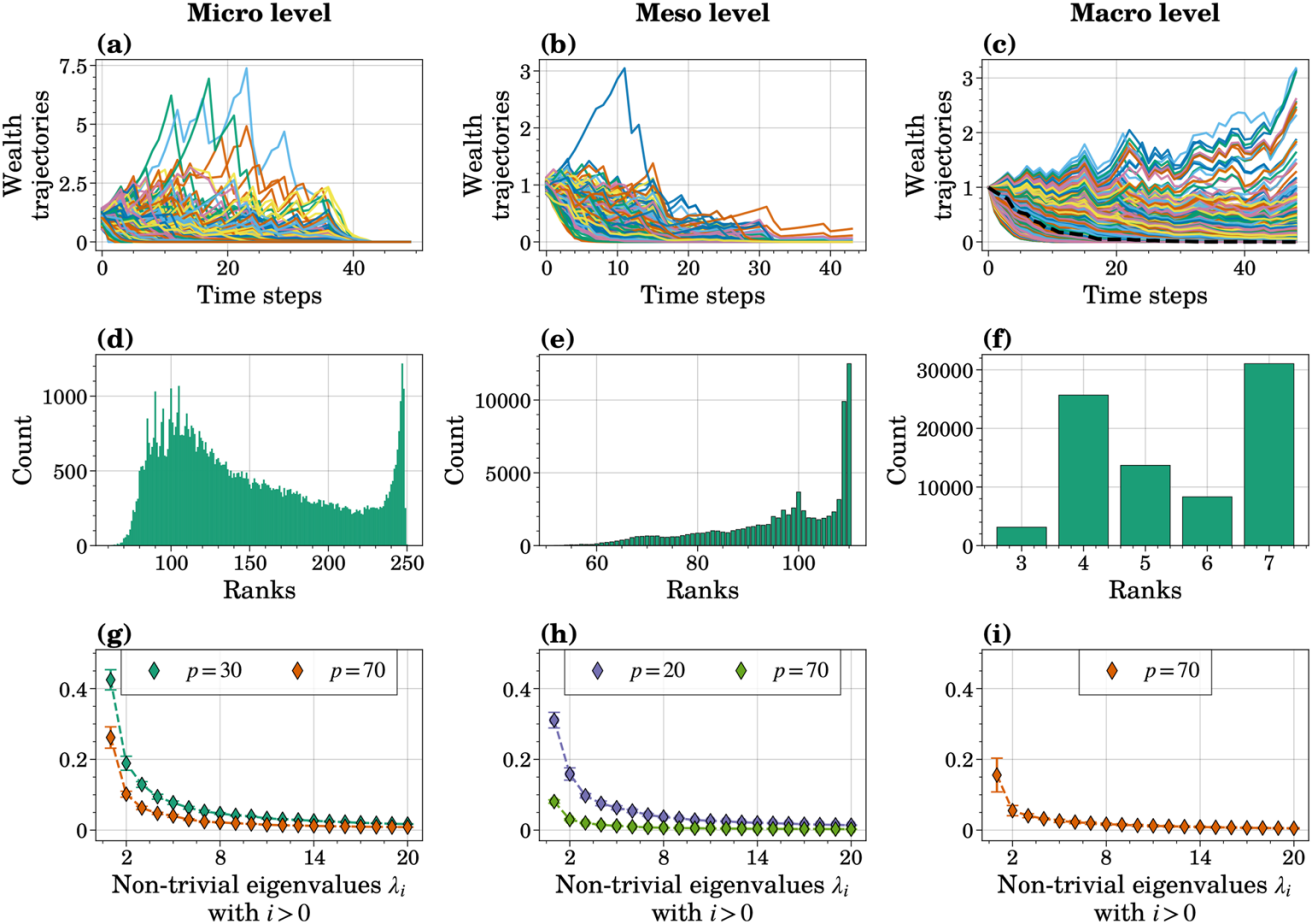
Preliminary analysis of the data from the poverty trap formation ABM with six uncertain input parameters at the micro, meso, and macro levels. (**a**,**b**) Trajectories at the micro and meso levels correspond to the same sample, $\varvec{X}_i$, in the experimental design, $\mathcal {X}$, and the same repetition (with a fixed random seed). (**c**) Each of the displayed 2048 macro-level trajectories (a subset of $N=8192$ trajectories) is related to a single combination of uncertain parameters in the experimental design for one run. The black dashed line corresponds to the average trajectory of individual agent trajectories in (**a**). (**d**–**f**) The frequency of rank occurrence in the entire data (10 repetitions) for the micro-, meso-, and macro-level data. (**g**–**i**) Scree-plots of mean eigenvalues from GDMaps on micro-, meso-, and macro-level outputs of the poverty trap formation ABM with six uncertain input parameters. Two Grassmannian dimensions, *p*, were investigated for the micro and meso levels, and one manifold dimension ($p=4$) was chosen for the macro level. Error bars indicate the variance across 10 runs.

Figure 5a–c display the wealth trajectories of individual agents, communities, and the entire population, respectively. A community trajectory is constructed as the average of constituent agent trajectories, while the population trajectories are the average trajectories of all agents for each combination of input parameter parameters. In the presented macro-level output, a number of agents surpass their initial wealth, yet all agents reach zero by the end of the simulation. The provided macro-level trajectories for all parameter combinations suggest that with certain parameter configurations, the final wealth of the population does not go to zero, indicating the presence of a double equilibrium poverty trap when a proportion of the population experiences relatively high levels of wealth compared to the rest of the population^7^.

Figure 5d–f present rank occurrence frequencies across all three levels after reshaping output matrices, as detailed in Methods. The most common ranks are either maximum or near-maximum ranks, with occasional other ranks appearing. We used these rank distributions to determine Grassmannian dimensions for each level. Thus, we chose $p=4$ for the macro level. Scree plots in Fig. 5g–i show spectral decays for all manifold dimensions, indicating dimensionality reduction efforts and suggesting that we could retain a substantial amount of the data variation with the first few non-trivial diffusion coordinates. We employed $p=70$ for the micro level and $p=20$ for the meso level for Sobol’ index calculations with a non-parsimonious representation since their eigenvalues are comparable. Supplementary Figs. S14 and S15 provide diffusion coordinates for one repetition at all levels.

**Figure 6:**
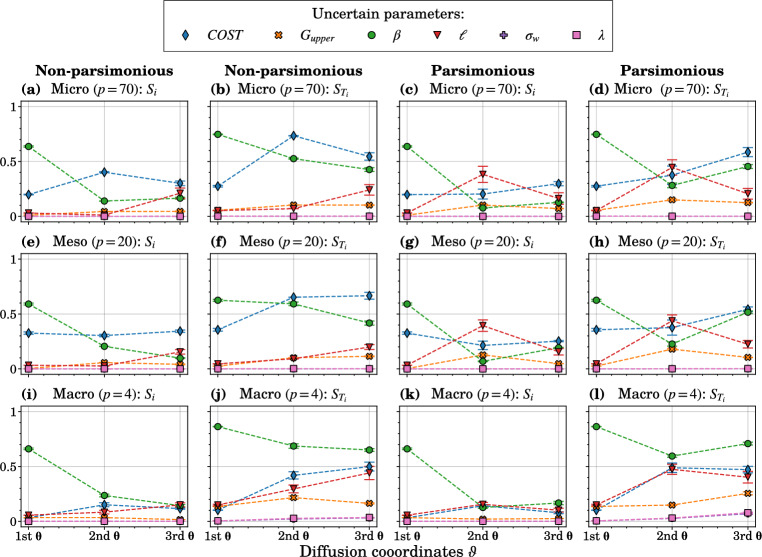
GSA results for six uncertain input parameters and three levels of the poverty trap formation ABM. The PCE coefficients used to compute Sobol’ indices were estimated using LAR. The results are plotted for the maximum polynomial degree of $s_{\max }=15$. Error bars indicate variance across 10 runs.

Figure 6 illustrates the resulting first- and total-order sensitivity indices obtained using LAR with $s_{\max }=12$, and the considered Grassmannian dimensions (other investigated maximum polynomial degrees, $s_{\max } \in \{3,6,9\}$, for the three levels as well as the error convergence are illustrated in Supplementary Figs. S16–S18).

At the macro level, interaction effects (except for $\beta$ for the first diffusion coordinates) are more pronounced, especially concerning the second and third diffusion coordinates, whether selected parsimoniously or non-parsimoniously. However, at the micro and meso levels, individual parameters play a more significant role, with minor differences. Across all levels and representation methods, the parameter $\beta$ consistently exhibits the highest Sobol’ indices for the first diffusion coordinates, which is expected as it controls savings for investments^7^. Noteworthy is the higher sensitivity of the *COST* parameter for the first diffusion coordinates at the meso level compared to the macro level, in line with its direct influence on meso-level dynamics as discussed by Dupont^7^.

An interesting finding is that the parameter $\ell$ yields the highest first- and total-order indices for the second $\varvec{\theta }$ at the micro and meso level and only for the parsimonious selection, highlighting the main difference between the two methods for selecting diffusion coordinates. This indicates that parsimonious selection identifies modes that correspond to the change in magnitude in the wealth trajectories, which is exerted by $\ell$. Similar sensitivity estimates across the micro and meso levels, with larger error bars for the former, can confirm this hypothesis, as meso-level data is constructed as community averages, thus still capturing the variability in wealth over time, which is influenced by $\ell$.

The utilization of the standard GSA technique in the context of the multi-level poverty model encounters challenges akin to those associated with temporal robustness and the dimensionality of the output space, at the macroscopic level. The computation of sensitivity indices at the micro and mesoscopic scales proves to be unfeasible through conventional techniques, as it necessitates the aggregation of agent-level data. A pivotal advantage inherent in our proposed approach lies in its capability to calculate sensitivity indices across the micro, meso, and macroscopic levels.

## Discussion

GSA is a widely employed method in diverse fields, including engineering, environmental science, and economics. Its purpose is to evaluate how input variability or uncertainty impacts a model’s output to identify the most influential input factors. GSA is guided by axioms that dictate the properties a good sensitivity index should ideally possess, including non-negativity, symmetry, range independence, decomposability, additivity, convergence, and moment-independence^9–11^. In this study, we went beyond traditional axioms and showed that a good sensitivity index should also be “temporally robust” and not depend on a specific snapshot of the model output. Specifically, we proposed a GSA approach using GDMaps PCE, which can aggregate entire trajectories and thus be particularly beneficial to study parametric sensitivities in models with non-linear behaviors, such as oscillations and regime shifts. In traditional time-dependent Sobol’ index estimation methods, it often becomes challenging to decisively discern which parameters exert relatively more influence and whether the variance in model output primarily stems from main effects or interaction effects. In contrast, our novel GSA approach allowed us to successfully reveal clear relations between parameters and their relative influence on the output variance. When we applied it to a large-scale spatial ABM of epidemic dynamics, it adeptly captured the strong interaction effects of uncertain parameters for multiple aggregated model trajectories.

Further, our GSA framework’s capability to handle high-dimensional model outputs allowed us to compute Sobol’ indices at the agent and community levels without additional modifications. The distinct temporal scales at which ABM agents operate, compared to the community and population-level processes, result in unique influences of uncertain parameters at these levels. We confirmed this hypothesis, revealing that indices at the micro and meso levels significantly differ from those at the macro level, which may be attributed to the relative richness of the reduced data preserved through higher-rank representations available at lower levels.

Interpreting sensitivity indices on the reduced space can be challenging, given the non-trivial task of making sense of diffusion coordinates associated with specific subspace structures identified by GDMaps. To address this, we related diffusion coordinates to their corresponding eigenvalues and leveraged physical interpretation of diffusion maps based on Markov Chain timescales. This interpretation provides insights into the influence of parameters on the model’s dynamics, with the first diffusion coordinates representing the slowest dynamics at a given level. As we move down the eigenvalue spectrum, diffusion coordinates capture more noise, especially those with significantly lower eigenvalues. These lower eigenvalues correspond to shorter-scale dynamics driven by interactions, which are useful to examine at different levels. For instance, our study revealed that for the second and third diffusion coordinates, first-order indices were higher at the micro and meso levels, indicating the significance of individual parameter effects on shorter-scale dynamics at these levels and underscoring that parameters can influence processes operating at various scales within the model.

We assessed the influence of key hyperparameters on sensitivity measures, namely the Grassmann manifold dimension and maximum polynomial degree used in a model selection technique based on LAR. Lower Grassmannian dimensions led to higher first-order indices, suggesting a coarser data representation. Optimizing embedding quality is a potential area for future research. We noticed error convergence and stable sensitivity estimates with larger degrees, indicating the effectiveness of sparse PCE in reducing overfitting. We also compared two methods for retaining diffusion coordinates, which mainly affected the second and third diffusion coordinates. The Grassmannian dimension and parsimonious representation emphasized specific dynamical modes and thus parameter sensitivities for the second and third diffusion coordinates.

The implications of this study are threefold. First, our trajectory-based approach addresses the limitations of conventional GSA approaches in capturing system dynamics, especially in models with non-linear behaviors, quantifying the influence input parameters have on system behavior. While we emphasize the framework’s suitability for ABMs, it can be applied to any data generation process, including various types of dynamic models. Second, considering the varying influences of uncertain parameters at different scales, studying parametric sensitivities at micro, meso, and macro levels is crucial for comprehension of ABM interactions exhibiting multi-scale dynamics. This GSA framework enriches the toolbox available to ABM practitioners, enabling them to analyze models with greater granularity and make more informed decisions during model design, calibration, and validation. Lastly, this study exhibits the utility of manifold learning techniques in capturing complex spatio-temporal processes. By demonstrating the versatility and successful applications of our method, we encourage researchers across diverse fields to harness manifold learning methods to gain deeper insights into a wide range of systems.

## Methods

In this section, we describe the standard approaches for computing Sobol’ indices as well as the proposed GSA framework, built upon our previous work^12^; the primary improvement is the use of sparse PCE, specifically, least angle regression (LAR), to calculate PCE coefficients.

### Variance-based global sensitivity analysis: Sobol’ indices

We consider a set of *d* independent random variables (RVs) $\varvec{X} = \{X_i \}_{i=1}^{d}$, serving as an input into a model $Y=\mathcal {M}(\cdot )$. For simplicity, we assume that the RVs $X_i$ are uniformly distributed on [0, 1]: $X_i \sim \mathcal {U}(0,1)$, $\Gamma = [0,1]^d$ and write the Sobol’ decomposition of the response $\mathcal {M}(\varvec{X})$ as the finite, hierarchical expansion:

1 $$\begin{aligned} \begin{aligned} \mathcal {M}(\varvec{X})&= \mathcal {M}_0 + \sum _{i=1}^d \mathcal {M}_i(X_i) + \sum _{i,j\ne i}^d \mathcal {M}_{ij}(X_i, X_j) + \dots + \mathcal {M}_{12\dots d}(\varvec{X}) \\&= \mathcal {M}_0 + \sum _{\varvec{u}\subset \{1, \dots , d\}} \mathcal {M}_{\varvec{u}}(\varvec{X}_{\varvec{u}}), \end{aligned} \end{aligned}$$

where $\varvec{X}_{\varvec{u}}:=\{X_{i_1}, \dots , X_{i_s}\}$ and the summands satisfy the orthogonality condition: $\int _{\Gamma } f_{\varvec{u}}(\varvec{X}_{\varvec{u}})f_{\varvec{v}}(\varvec{X}_{\varvec{v}})d\varvec{X} = 0 \quad \forall \varvec{u}\ne \varvec{v}$. In Equation 1, $\mathcal {M}_0$ is the mean response of $\mathcal {M}$, the univariate functions $\mathcal {M}_i(X_i)$ quantify independent contributions given the individual parameters, the bivariate functions $\mathcal {M}_{ij}(X_i, X_j)$ represent the effect of interactions between $X_i$ and $X_j$ on the response, with similar interpretations for higher-order interaction effects^13^.

From the total variance theorem, the total variance $\mathbb {V}[Y] = D$ can be decomposed as

2 $$\begin{aligned} D = \sum _{i=1}^d D_i + \sum _{1\le i<j\le d}D_{ij} + \dots + D_{12\dots d}, \end{aligned}$$

which we use to define first- and total-order Sobol’ indices as

3 $$\begin{aligned} S_i = \frac{D_i}{D} = \frac{\mathbb {V}[\mathbb {E}(Y|X_i)]}{\mathbb {V}[Y]}, \quad S_{T_i} = 1 - \frac{D_{\sim i}}{D}=1-\frac{\mathbb {V}[\mathbb {E}(Y|\varvec{X}_{\sim i})]}{\mathbb {V}[Y]} = \frac{\mathbb {E}[\mathbb {V}(Y|\varvec{X}_{\sim i})]}{\mathbb {V}[Y]}. \end{aligned}$$

#### Calculation of Sobol’ indices with conventional methods

Using Monte-Carlo (MC), we obtain the following estimators for mean as

4 $$\begin{aligned} \widehat{\mathcal {M}}_0 = \frac{1}{N}\sum _{n=1}^N\mathcal {M}(\varvec{X}^{(n)}), \end{aligned}$$

and for total variance as

5 $$\begin{aligned} \widehat{D} = \frac{1}{N}\sum _{n=1}^N\mathcal {M}^2(\varvec{X}^{(n)}) - \widehat{\mathcal {M}}^2_0, \end{aligned}$$

where $\varvec{X}^{(n)}$ denoted independent and identically distributed (iid) samples of $\varvec{X}$. To obtain the estimates for $D_i$ and $D_{\sim i}$, we use Saltelli’s algorithm (explained in^13,14^) to reduce the number of evaluations from $N^2$ for crude MC to $N(d+2)$ by constructing three types of samples: $\varvec{X}=(X_1, \dots , X_d)^{\top }$, its complete resample $\varvec{X}' = (X'_1, \dots , X'_d)^{\top }$, and $(X_i, \varvec{X}'_{\sim i})=(X'_1, \dots , X'_{i-1}, X_i, X'_{i+1}, \dots , X'_d)^{\top }$, with $i = 1, \dots , d$, where all factors except for $X_i$ are resampled. Thus, the estimates for $D_i$ and $D_{\sim i}$ become

6 $$\begin{aligned} \widehat{D}_i = \frac{1}{N}\sum _{n=1}^N\mathcal {M}(X_i^{(n)}\varvec{X}_{\sim i}^{(n)})\mathcal {M}(X_i^{(n)}{\varvec{X}'}_{\sim i}^{(n)}) - \widehat{\mathcal {M}}^2_0 \end{aligned}$$

and

7 $$\begin{aligned} \widehat{D}_{\sim i} = \frac{1}{N}\sum _{n=1}^N \mathcal {M}(X_i^{(n)}{\varvec{X}'}_{\sim i}^{(n)}) \mathcal {M}({X'}_i^{(n)}{\varvec{X}'}_{\sim i}^{(n)}) - \widehat{\mathcal {M}}^2_0, \end{aligned}$$

respectively. The derived estimators $\widehat{D}$, $\widehat{D}_i$ and $\widehat{D}_{\sim i}$ are then used to calculate the first- and total-order Sobol’ indices in Equation 3.

#### Sobol’ indices using PCE

Polynomial chaos expansion (PCE) is a mathematical technique that describes the input-output relationship of a model or a function using polynomials orthogonal with respect to the probability density function (PDF) of the input RVs. The choice of polynomials is based on the probability distribution of the uncertain parameters. For example, Hermite polynomials are used for Gaussian distributions, Legendre polynomials for uniform distributions, and so on^15^. Sobol’ decomposition of a PCE results from reordering terms of the truncated PCE, defined as

8 $$\begin{aligned} \mathcal {M}(\varvec{X}) \approx \widetilde{\mathcal {M}}(\varvec{X}) = \sum _{\varvec{\alpha } \in \mathbf {\mathcal {A}}} c_{\varvec{\alpha }} \Psi _{\varvec{\alpha }}(\varvec{X}), \end{aligned}$$

where $\mathbf {\mathcal {A}}$ is a total-degree multi-index set with $\varvec{\alpha }$ being equivalent to the multivariate polynomial degree, $c_{\varvec{\alpha }}$ as the corresponding PCE coefficients, and $\Psi _{\varvec{\alpha }}(\textbf{X})$ as the multivariate orthonormal polynomials with respect to PDF $f_{\textbf{X}}$ such that $\langle \Psi _{\varvec{\alpha }}(\varvec{X}),\Psi _{\varvec{\beta }}(\varvec{X}) \rangle = \int _{\Gamma } \Psi _{\varvec{\alpha }}(\varvec{X})\Psi _{\varvec{\beta }}(\varvec{X})f_{\varvec{X}}(\varvec{X})d\varvec{X} = \gamma _{\varvec{\alpha }}\delta _{\varvec{\alpha \beta }}$, where $\delta _{\varvec{\alpha \beta }}$ denotes the Kronecker delta and $\gamma _{\varvec{\alpha }}$ is the normalization factor. The mean and variance of $\widetilde{\mathcal {M}}(\varvec{X})$ can be respectively approximated using

9 $$\begin{aligned} \mathbb {E}[\widetilde{\mathcal {M}}(\varvec{X})] = c_{\varvec{0}}, \quad \text {and}\quad \mathbb {V}[\widetilde{\mathcal {M}}(\varvec{X})] = \sum _{\varvec{\alpha } \in \mathbf {\mathcal {A}}\backslash \varvec{0}} c^2_{\varvec{\alpha }}. \end{aligned}$$

To calculate sensitivity indices, we require partial variances next to the variance expression in Equation 9. For this purpose, we obtain interaction sets as $\mathcal {A}_{\varvec{u}} = \{\varvec{\alpha } \in \mathcal {A}: i \in \varvec{u} \Leftrightarrow \alpha _{i} \ne 0 \}$ for a given $\varvec{u} := \{i_1, \dots , i_s\}$, leading to the following decomposition: $\mathcal {M}(\varvec{X}) = \mathcal {M}_0 + \sum _{\varvec{u}\subset \{1, \dots , d\}} \mathcal {M}_{\varvec{u}}(\varvec{X}_{\varvec{u}})$, with $\mathcal {M}_{\varvec{u}}(\varvec{X}_{\varvec{u}}) := \sum _{\varvec{\alpha } \in \mathbf {\mathcal {A}}_{\varvec{u}}} c_{\varvec{\alpha }} \Psi _{\varvec{\alpha }}(\varvec{X})$. Hence, we get the following general expression for PCE-based Sobol’ indices, which can be derived analytically at any order from the PCE coefficients^16^:

10 $$\begin{aligned} S_{\varvec{u}} = \frac{D_{\varvec{u}}}{D} = \frac{\sum _{\varvec{\alpha } \in \mathbf {\mathcal {A}}_{\varvec{u}}}c^2_{\varvec{\alpha }}}{\sum _{\varvec{\alpha } \in \mathbf {\mathcal {A}}\backslash \varvec{0}}c^2_{\varvec{\alpha }}}. \end{aligned}$$

### GSA using Grassmannian diffusion maps and PCE

The methodology outlined in this section is largely based on GDMaps as proposed by Dos Santos et al.^17^ and manifold learning-based PCE developed by Kontolati et al.^5^. We refer readers to the corresponding papers for a thorough description of the Grassmann manifold principles and other elements of differential geometry essential for developing GDMaps. An intuitive explanation of the Grassmann manifold and some crucial concepts from the algebraic geometry can be found in the SI, Sect. S5. It is important, however, for its later use to provide here a definition for the Grassmann manifold, or Grassmannian, denoted as $\mathcal {G}(p, n)$, as a set of *p*-dimensional subspaces (or *p*-planes) embedded in *n*-dimensional Euclidean space, $\mathbb {R}^n$. Details on the implementation of the framework are summarized in Algorithm 2.

#### Grassmannian diffusion maps

GDMaps is a two-stage dimensionality reduction technique involving linear pointwise and non-linear multipoint dimension reduction. We start with an ensemble of *N* independent and identically distributed samples drawn from a joint PDF, $f_{\varvec{X}}$, comprising experimental design $\mathcal {X}=(\varvec{X}_i)_{i=1}^N, \varvec{X}_i \in \mathbb {R}^k$ and *N* high-dimensional output data produced by a model (e.g., an ABM), $\mathcal {Y}=(\mathcal {M}(\varvec{X}_i))_{i=1}^{N} = (\varvec{Y}_i)_{i=1}^{N}, \varvec{Y}_i \in \mathbb {R}^{n \times m}$.

The first step is to project each data point, $\varvec{Y}_{i}$, onto a Grassmann manifold, assuming that $\varvec{Y}_{i}$ has a low-rank structure (i.e., the number of linearly independent columns is lower than the total number of columns). The projection is performed via the singular value decomposition (SVD) for each point, $\varvec{Y}_{i}$, into three matrices:

11 $$\begin{aligned} \varvec{Y}_{i} = \varvec{U}_i\varvec{\Sigma }_i\varvec{V}_i^{\top }, \end{aligned}$$

where $\varvec{\Sigma }_{i} \in \mathbb {R}^{p \times p}$ is a diagonal matrix containing singular values. $\varvec{U}_{i} \in \mathbb {R}^{n \times p}$ and $\varvec{V}_{i} \in \mathbb {R}^{m \times p}$ are orthonormal matrices; hence, we consider them to reside on the Grassmannian manifolds: $\mathcal {G}(p, n)=\{\text {span}(\varvec{U}): \varvec{U} \in \mathbb {R}^{n \times p}\}$ and $\mathcal {G}(p, m)=\{\text {span}(\varvec{V}): \varvec{V} \in \mathbb {R}^{m \times p}\}$, respectively. The number of *p*-planes defining the Grassmann manifold is an important hyper-parameter determining the ranks of $\varvec{U}_i$ and $\varvec{V}_i$, and thus the accuracy level of data representation on the Grassmannian, which was found to significantly affect the predictive ability of a surrogate constructed at a later stage^5^. The Grassmannian dimension, *p*, can be specified a priori, e.g., as a maximum or minimum rank of all input matrices, $\varvec{Y}_{i}$. Practically speaking, we use SVD to identify a basis for each $\varvec{Y}_i$, and the resulting space spanned by this basis is a point on the Grassmannian representing the projection of the data onto the manifold. $\varvec{U}_i$ and $\varvec{V}_i$ represent the dimension-reduced data on which we later operate. We can connect it to the idea of operating on a propagator, as discussed in SI, Sects. S2 and S3.

We begin the non-linear dimensionality reduction step by considering a positive semi-definite Grassmannian kernel defined as a map, $k: \mathcal {G}(p, n) \times \mathcal {G}(p, n) \rightarrow \mathbb {R}$, and construct the kernel matrices, $[k(\varvec{U}_{i}, \varvec{U}_{j})] \in \mathbb {R}^{N \times N}$ and $[k(\varvec{V}_{i}, \varvec{V}_{j})] \in \mathbb {R}^{N \times N}$. In this work, we use the projection kernel defined as

12 $$\begin{aligned} k_{\text {pr}}(\varvec{U}_{i}, \varvec{U}_{j})=\left\| \varvec{U}_{i}^{T} \varvec{U}_{j}\right\| _{F}^{2}, \end{aligned}$$

where *F* denotes the Frobenius norm (i.e., the Euclidian norm of a matrix). For further information about other Grassmannian kernels, their construction and application, we refer the reader to Dos Santos et al.^17^. Using the constructed kernels, we compile the composed kernel matrix, $K(\varvec{U}, \varvec{V})$, by taking the Hadamard product of the corresponding kernels: $K(\varvec{U}, \varvec{V}) = K(\varvec{U}) \circ K(\varvec{V})$. Alternatively, one can create the composed kernel matrix by summing $K(\varvec{U}) + K(\varvec{V})$.

The composed kernel matrix is used to define a random walk, $W=(\{S_{\mathcal {U}}, S_{\mathcal {V}}\}, f, \varvec{P})$, with probability distribution, *f*, and transition probability matrix, $\varvec{P}$, over the data on the Grassmann manifold, given by sets $S_{\mathcal {U}}=\{\mathcal {U}_i\}_{i=1}^{N}$ and $S_{\mathcal {V}}=\{\mathcal {V}_i\}_{i=1}^{N}$, where $\mathcal {U}_i = \text {span}(\varvec{U}_i)$ and $\mathcal {V}_i = \text {span}(\varvec{V}_i)$, respectively. The transition probability matrix, $\varvec{P} = [P_{ij}]$, of the random walk, *W*, over the Grassmannian is built as follows. First, the diagonal degree matrix, $\varvec{D} \in \mathbb {R}^{N \times N}$, is constructed as $D_{ii} = \sum _{j=1}^{N}k_{ij}$, thereby determining the stationary distribution of the random walk, *W*, as $\pi _i = D_{ii}/\left( \sum _{k=1}^{N}D_{kk}\right)$. Next, we use the kernel matrix, $k_{ij}$, normalized as $\kappa _{ij} = k_{ij}/\sqrt{D_{ii}D_{jj}}$ to obtain the transition probability matrix, $\varvec{P}=[P_{ij}]$, given by

13 $$\begin{aligned} P_{ij} = \frac{k_{ij}}{\sum _{k=1}^{N}\kappa _{ik}}. \end{aligned}$$

Thus, we run the diffusion process on the manifold, and from the eigendecomposition of $\varvec{P}$, we retain the first *g* eigenvectors $\{\varvec{\xi }_j \in \mathbb {R}^{N} \}_{j=1}^g$, (note that the index starts from $j=1$, as diffusion coordinates corresponding to trivial eigenvalue, $\lambda _0 = 1$, can be omitted), and their respective eigenvalues, $\{\lambda _i\}_{j=1}^g$. The Grassmannian diffusion coordinates for every element, $\varvec{Y}_i \in \mathcal {Y}$, are calculated as

14 $$\begin{aligned} \varvec{\Theta }_{l} = (\theta _{l1}, \dots , \theta _{lg}) = (\lambda _1\xi _{l1}, \dots , \lambda _g\xi _{lg}). \end{aligned}$$

Given the decaying spectrum of eigenvalues, $\{\lambda _0, \dots , \lambda _{g}\}$, of the sparse Markov matrix, it is sufficient to keep a small number, *g*, of diffusion coordinates to describe the essential geometric structure of the data^5^. We use two notations to refer to diffusion coordinates: sequence, $\Theta = (\varvec{\Theta }_i)_{i=1}^{N}, \Theta _i \in \mathbb {R}^g$ and set, $\vartheta = \{\varvec{\theta }_j \in \mathbb {R}^{N}\}_{j=1}^g$. We employ the first notation to define a map between samples in the experimental design and the diffusion space and use the second notation to refer to the first *g* diffusion coordinates of length *N*, either parsimoniously selected (see^8^) or corresponding to the largest *g* eigenvalues.

#### Polynomial chaos expansion on a low-dimensional manifold

PCE expresses the relationship between the input and output in terms of orthogonal polynomials chosen to be orthonormal with respect to the PDF of the input random variables. Here, the input is defined as $\mathcal {X}=(\varvec{X}_i)_{i=1}^N, \varvec{X}_i \in \mathbb {R}^k$ with assumed independent components described by a joint probability distribution, $f_{\varvec{X}}$, and the output is diffusion coordinates, representing the low-dimensional manifold of the original data, $\Theta =(\varvec{\Theta }_i)_{i=1}^N, \varvec{\Theta }_i \in \mathbb {R}^k$. Thus, we use a PCE surrogate model to approximate a mapping between input parameters and coordinates on the diffusion manifold, $\mathcal {E}: \varvec{X} \rightarrow \varvec{\Theta }$ as:

15 $$\begin{aligned} \mathcal {E}(\varvec{X}) \approx \widetilde{\mathcal {E}}(\varvec{X})=\sum _{\varvec{s} \in \Lambda } \varvec{c}_{\varvec{s}} \Psi _{\varvec{s}}(\varvec{X}), \end{aligned}$$

where $\Psi _{\varvec{s}}(\varvec{X}) = \prod _{i=1}^{k}\psi _{s_i}^{(i)}(X_i)$, assuming independence, are multivariate orthonormal polynomials with respect to $f_{\varvec{X}}$, satisfying the orthogonality condition, $\langle \Psi _{\varvec{s}}(\varvec{X}),\Psi _{\varvec{t}}(\varvec{X}) \rangle = \int _{\Gamma } \Psi _{\varvec{\alpha }}(\varvec{X})\Psi _{\varvec{\beta }}(\varvec{X})f_{\varvec{X}}(\varvec{X})d\varvec{X} = \gamma _{\varvec{s}}\delta _{\varvec{st}}$, where $\delta _{\varvec{st}}$ denotes the Kronecker delta and $\gamma _{\varvec{s}}$ is the normalization factor; $\varvec{c}_{\varvec{s}} \in \mathbb {R}^g$ are corresponding PCE coefficients that are vector-valued with dimension equal to the number of retained diffusion coordinates, *g*. Multi-indices, $\varvec{s} = (s_1, \dots , s_k)$, are uniquely associated with the single indices, *s*, and $\Lambda$ is a total-degree multi-index set satisfying $\Vert \varvec{s}\Vert _{1} \le s_{\max }, s_{\max } \in \mathbb {Z}_{\ge 0}$, resulting in a PCE basis of size, $\frac{(k+s_{\max })!}{k!s_{\max }!}$, which scales polynomially with the number of input dimensions, *k*, and maximum polynomial degree, $s_{\max }$.

With the fixed multi-index set, $\Lambda$, one can get PCE coefficients, $\varvec{c}_{\varvec{s}}$, by solving the ordinary least squares (OLS) problem:

16 $$\begin{aligned} \underset{\varvec{c} \in \mathbb {R}^{|\Lambda |}}{\arg \min }\frac{1}{N} \sum _{i=1}^{N}\left( \mathcal {E}(\varvec{X}_i )-\sum _{\varvec{s} \in \Lambda } \varvec{c}_{\varvec{s}}\Psi _{\varvec{s}}(\varvec{X}_i)\right) ^2. \end{aligned}$$

When employing OLS for the calculation of PCE coefficients, the number of functions, *P*, in a PCE basis is calculated as $(k+s_{\max })!/(k!s_{\max }!)$, thus requiring the size, *N*, of the experimental design, $\mathcal {X}$, to be $N \ge P$ for the problem to be well-conditioned (practically, the size of experimental design is often put to $N = dP$, where $d \in \{2,3\}$)^18–20^, suggesting a requirement for a large experimental design.

To remedy the issue of polynomial scaling of the number of basis function, *P*, we utilize an adaptive model selection algorithm for sparse PCE-based on least angle regression (LAR)^20^. Introduced by Efron et al.^21^, LAR is used in statistics and machine learning to select important variables or features from a larger set when building a model. Intuitively, the LAR algorithm proceeds as follows: the model begins with no predictors; it selects a predictor most correlated with the residual and moves in its direction until another nonactive regressor becomes equally correlated with the residual; then the algorithm shifts along a direction equiangular to both predictors^22^. This process continues until all predictors are included in the model. Additionally, a sequence of sets containing indices of active basis functions is maintained to track included predictors, with the algorithm progressing iteratively to build the model. This sequence can be written as $\Lambda _1 \subset \Lambda _2 \subset \cdots \Lambda _m$, where $m = \min (P, N)$^22^.

In the hybrid LAR procedure, LAR is employed first to select a set of predictors, and the corresponding coefficients are then computed with OLS on the selected basis, $\Lambda _i$^20,21^. This combination of two methods allows for a minimal error for each metamodel, $(\Lambda _i, \varvec{c}_i)$, where $\varvec{c}_i$ denotes corresponding coefficients^22^. An optimal metamodel (i.e., optimal sparsity level) can be found by assessing a model selection criterion of choice, for instance, the leave-one-out (LOO) error, defined as

17 $$\begin{aligned} \epsilon _{\text {LOO}} = \frac{\mathbb {E}\left[ \left( \Delta _i / (1 - h_i)\right) ^2\right] }{\mathbb {V}[Y]}, \end{aligned}$$

where $\mathbb {V}[Y]$ is the variance of model response, $h_i$ is the *i*-th diagonal term of the matrix $\varvec{\Phi }(\varvec{\Phi }^{\top }\varvec{\Phi })^{-1}\varvec{\Phi }^{\top }$ ($\varvec{\Phi }$ here is the design matrix) and $\Delta _i$ is the difference between the model evaluation at $\varvec{x}^{(i)}$ and its approximation:

18 $$\begin{aligned} \Delta _i = \mathcal {M}(\varvec{x}^{(i)}) - \widetilde{\mathcal {M}}(\varvec{x}^{(i)}). \end{aligned}$$

Hybrid LAR can be used to adaptively construct a PCE surrogate for a given experimental design, as presented in Algorithm 1. Note that the maximum target error is $\epsilon _{\text {target}}=1$, and when it is set close to this value, there exists a risk of overfitting. Hence, the following condition for overfitting is introduced: if three consecutive steps result in decreased accuracy, the algorithm stops.

Using Algorithm 1, we can now compute sparse PCE coefficients, $\varvec{c}_{\text {opt}}$, of the surrogate model on the low-dimensional manifold obtained with GDMaps and estimate Sobol’ indices without additional computational cost. In the context of computing PCE coefficients, it is important to note that with OLS, coefficients can be computed for all *g* diffusion coefficients simultaneously. However, when using LAR, one must iterate through the *g* diffusion coefficients and perform the adaptive procedure individually for each $\varvec{\theta }_i$ as an optimal basis can be dissimilar for different data, $(\mathcal {X}, \varvec{y})$.

**Algorithm 1:**
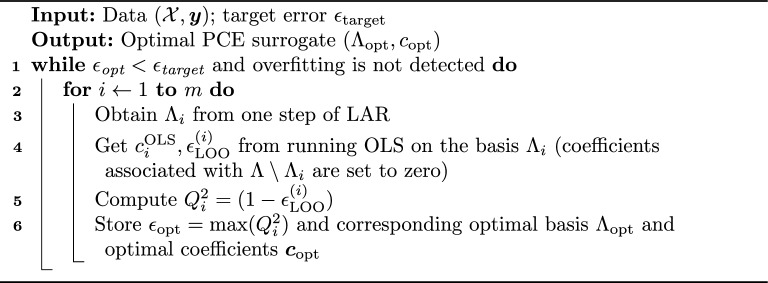
Best model selection algorithm based on hybrid LAR^20,21^.

To approximate generalization error, we use the validation error, as defined by Kontolati et al.^5^:

19 $$\begin{aligned} \epsilon _{\textrm{val}}=\frac{\sum _{i=1}^{N_{*}}\left( \varvec{\Theta }_{i}^{*}-\widetilde{\mathcal {E}}\left( \varvec{X}_{i}^{*}\right) \right) ^2}{\sum _{i=1}^{N_{*}}\left( \varvec{\Theta }_{i}^{*}-\bar{\varvec{\Theta }}^{*}\right) ^{2}}, \end{aligned}$$

where $(\varvec{X}_{i}^{*})_{i=1}^{N_{*}}, \varvec{X}_{i}^{*} \in \mathbb {R}^{k}$ and $(\varvec{\Theta }_{i}^{*})_{i=1}^{N_{*}}, \varvec{\Theta }_{i}^{*}\in \mathbb {R}^{g}$ constitute a test set, which we choose to be of size $N_{*} = \frac{1}{3}N$ and $\bar{\varvec{\Theta }}^{*}=\frac{1}{N_{*}} \sum _{i=1}^{N_{*}} \varvec{\Theta }_{i}^{*}$ is the mean response of the test set on the latent space. Additionally, we assess the LOO error $\epsilon _{\text {LOO}}$ as defined in Eq. (17). We can also consider mean absolute error (MAE) given by:

20 $$\begin{aligned} \text {MAE} = \frac{\sum _{i=1}^{N_{*}}| \varvec{\Theta }_{i}^{*}-\widetilde{\mathcal {E}}\left( \varvec{X}_{i}^{*}\right) |}{N_{*}}. \end{aligned}$$

#### Sobol’ indices using polynomial chaos expansion on a low-dimensional manifold

Necessary components for calculating Sobol’ indices include the total variance, $D = \mathbb {V}[Y]$, and the partial variances of individual parameters and their interactions. Once the coefficients, $\varvec{c}_{\varvec{s}}$, in Eq. (15) have been determined, it becomes straightforward to obtain estimates for the Sobol’ indices. That is, the mean and variance of $\widetilde{\mathcal {E}}(\textbf{X})$ can be easily approximated using

21 $$\begin{aligned} \mathbb {E}[\widetilde{\mathcal {E}}(\varvec{X})] = \varvec{c}_{\varvec{0}} \quad \text {and}\quad \mathbb {V}[\widetilde{\mathcal {E}}(\varvec{X})] = \sum _{\varvec{\alpha } \in \mathbf {\mathcal {A}}\backslash \varvec{0}} \varvec{c}^2_{\varvec{\alpha }}. \end{aligned}$$

Regarding the partial variances, we can interpret the PCE coefficients with the multi-index set, $\Lambda$, as partial variances, provided that specific interactions are already determined by multi-indices, $\varvec{s}$. In particular, each multi-index, $\varvec{s} = (s_1,..., s_k)$, is uniquely characterized by single indices, *s*, corresponding to each of the *k* random inputs. As a result, we can gather multi-indices related to partial variance caused by random inputs indexed by *i*, individually (i.e., first-order effects) or in conjunction with the remaining random inputs (i.e., total-order effects), into two multi-index sets: $\Lambda _i = \{\varvec{s} \in \Lambda : s_i \ne 0, s_l = 0, \forall l \ne i\}$ and $\Lambda _{T_i} = \{\varvec{s} \in \Lambda : s_i \ne 0\}$, where $\Lambda _i \subset \Lambda$ and $\Lambda _{T_i} \subset \Lambda$. Given that the PCE coefficients are vector-valued with the dimension equal to the number of the retained diffusion coefficients, i.e., $\varvec{c}_{\textbf{s}} \in \mathbb {R}^g$, the corresponding first- and total-order Sobol’ indices are estimated as

22 $$\begin{aligned} \varvec{S}_{i} = \frac{\sum _{\varvec{s} \in \Lambda _{i}}\varvec{c}^2_{\varvec{s}}}{\sum _{\varvec{s} \in \Lambda \backslash \varvec{0}}\varvec{c}^2_{\varvec{s}}} \quad \text {and} \quad \varvec{S}_{T_{i}} = \frac{\sum _{\varvec{s} \in \Lambda _{T_i}}\varvec{c}^2_{\varvec{s}}}{\sum _{\varvec{s} \in \Lambda \backslash \varvec{0}}\varvec{c}^2_{\varvec{s}}}. \end{aligned}$$

The resulting sensitivity indices are thus vector-valued, with dimension $\in \mathbb {R}^{g}$, given that the dimension of the retained diffusion coefficients is *g*.

**Algorithm 2:**
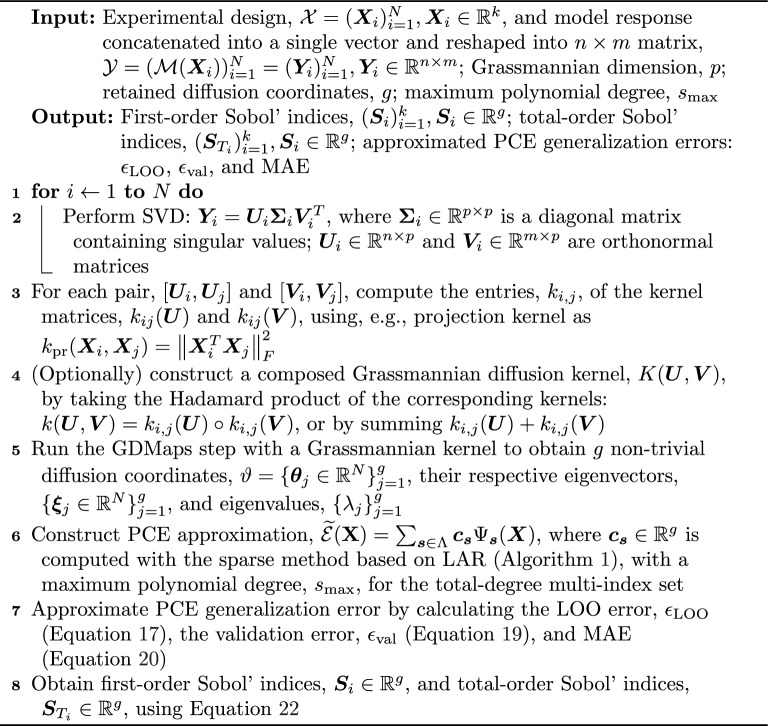
GSA using a manifold learning-based PCE based on^12^.

### Applications

We chose three different models to showcase the versatility of the framework. The first one is a classic dynamical system known as the Lotka–Volterra model^5^, while the other two are ABMs—DeepABM-COVID^6^ and a poverty trap formation ABM^7^. We used the Lotka–Volterra model to indirectly compare the performance of our GSA framework with the traditional Sobol’ index calculation methods across multiple time steps. The DeepABM-COVID model illustrated how the framework can be applied to analyze aggregated trajectories of multiple dependent model responses, while the poverty trap formation ABM was employed to investigate parametric sensitivities at various levels of analysis.

#### Lotka–Volterra dynamical system

The first model considered for applying the GSA framework using GDMaps PCE was the classic Lotka–Volterra dynamical system, used to model population dynamics arising from interactions between a prey species and a predator species. The model is defined in the following system of two coupled non-linear ordinary differential equations:

23 $$\begin{aligned} \begin{aligned} \frac{du}{dt}&= \alpha u - \beta uv, \\ \frac{dv}{dt}&=\delta uv - \gamma v, \end{aligned} \end{aligned}$$

where $u \ge 0$ is the prey population, $v \ge 0$ is the predator population, and $\alpha ,\beta ,\gamma ,\delta$ are stochastic model parameters described in Table 1.

GSA was applied to the Lotka–Volterra model with two uncertain parameters following an example from Kontolati et al. Note that the distributions of parameters in the thesis differ slightly from those in the reference paper. The distribution ranges used in this thesis follow those used in the code accompanying^5^. The initial conditions for prey and predator populations were $u(t = 0) = 10$ and $v(t = 0) = 5$, respectively; the distributions of uncertain parameters ($\alpha$ and $\beta$) and values for fixed parameters ($\gamma$ and $\delta$) followed Table 1. The accuracy of the surrogate model using GDMaps PCE was tested by Kontolati et al.^5^ through out-of-sample reconstruction, which ensured that GSA applied on the surrogate would also result in accurate estimates. Following the best-performing sample size from the reference example, $N = 600$ samples ($\mathcal {X} \in \mathbb {R}^{600 \times 2}$) were randomly generated. As in the reference, for each combination of two parameters in the sample, the system was solved using a time period of $T=25$, discretized using $n = 512$ points. The output was a $\mathcal {Y} \in \mathbb {R}^{600 \times 1024}$ matrix, where solutions, $\{u(t), v(t)\}$, for each corresponding sample were concatenated into a single vector. Each of the 600 solutions was reshaped to a square matrix, $\varvec{Y}_i \in \mathbb {R}^{32 \times 32}$. Performing GDMaps with a constant value of $p = 10$ yielded orthonormal matrices on the Grassmannian, $\{\varvec{U}_i, \varvec{V}_i \in \mathcal {G}_{(10, 32)}\}_{i=1}^{N=600}$. We preserved three parsimoniously selected diffusion coordinates ($g = 3$) and employed a PCE surrogate that utilized a total-degree basis with a maximum polynomial degree of $s_{\max } = 6$. To calculate the first- and total-order sensitivity indices, we resampled the parameters 50 times and averaged the resulting GSA estimates over the repeated surrogates. We contrasted the proposed GSA method with the conventional calculation of Sobol’ sensitivity indices by obtaining the first and total order indices separately for two model outputs, *u* and *v*, for 15 evenly spaced time steps for both setups.

**Table 1:** Details of the uncertain input parameters for the Lotka–Volterra dynamical system.

Variable	Distribution	Description
$\alpha$	$\sim \mathcal {U}(0.90, 1.05)$	Natural growth rate of prey in the absence of predation
$\beta$	$\sim \mathcal {U}(0.10, 0.18)$	Death rate per encounter of prey due to predation
$\gamma$	1.50	Natural death rate of predators in the absence of prey
$\delta$	0.75	Reproduction rate of predators per prey eaten

#### DeepABM-COVID

The second model used to test the GSA method employing GDMaps PCE was DeepABM-COVID. Using the DeepABM framework, this model leverages the concepts of tensor-based calculus and graph neural networks to efficiently execute ABMs on GPU architectures and scale them to population-representative sizes^6^. The efficiency and speed of simulations for a large-scale spatial model were the main reasons for choosing the DeepABM framework to evaluate the GSA framework using GDMaps PCE. In the framework, agents are represented not as objects but as agent states organized as tensors. Interactions between agents are expressed as permutation-invariant message-passing processes in graph neural networks. Utilizing this framework, DeepABM-COVID simulates COVID-19 spread and transmission dynamics across a default of 100,000 agents for 180 time steps. Additional settings enable researchers to investigate the influence of various interventions such as quarantine, exposure notification, testing, and vaccination. For testing the proposed GSA framework, however, all intervention strategies were excluded from simulations to reduce the number of uncertain parameters. Thus, the model components related to interventions are not discussed here.

All agent states are comprised of static and dynamic components in the model. As the name suggests, the static component stays unchanged throughout a simulation while affecting an agent’s interactions with other agents. Agents’ static components incorporate the following categorical variables: (1) age, (2) occupation, (3) household, and (4) the number of daily interactions. Each agent is initialized with these attributes based on real-world census data (for attributes 1–3) and mobility data (for attribute 4) specific to King’s County in the State of Washington. Excluding the model elements corresponding to intervention strategies, the dynamic component changing over time and influencing model events incorporates the current disease stage, which at any step takes one of 11 predefined values: “susceptible”, “asymptomatic”, “presymptomatic mild”, “presymptomatic severe”, “mild-symptomatic”, “severe symptomatic”, “hospitalized”, “critical in ICU”, “death”, “hospitalized recovering”, “recovered”.

In each interaction, the transmission of the pathogen in DeepABM-COVID is modelled as follows:

24 $$\begin{aligned} P(t, s_i, a_s, n) = 1 - e^{-\lambda (t, s_i, a_s, n)}, \end{aligned}$$

where the $\lambda$-function in the exponent is defined as:

25 $$\begin{aligned} \lambda (t, s_i, a_s, n) = \frac{R S_{a_s}A_{s_i}B_n}{\bar{I}}\int _{t-1}^t f_{\Gamma }(u;\mu _i, \sigma _i^2)du. \end{aligned}$$

In the expressions above, *t* is the time elapsed since infection; $s_i$ denotes the symptom status of an infected individual (asymptomatic, mild/severe); $a_s$ indicates the age of the susceptible agents; and *n* is the interaction network type. The following parameters influence the viral transmission: *R*, a scalar for the overall infection rate using the simplified assumption that it is the mean number of individuals infected by a mildly/severely symptomatic person; $S_{a_s}$, the scale-factor for the age of the susceptible under the assumption that some age groups are more susceptible to the pathogen than others; $A_{s_i}$, the scalar for an infected individual being asymptomatic; $B_n$, the scale-factor for the interaction network accounting for the duration of interaction; $\bar{I}$, the mean number of daily interactions (see static parameters), and $f_{\Gamma }(u;\mu _i, \sigma _i^2)$, the PDF of a gamma distribution corresponding to the infection duration. Accordingly, the rate of infection transmission depends on (1) the infectivity of the pathogen, (2) the age-dependent susceptibility level, (3) the age-dependent level of being asymptomatic, and (4) the type of interaction network (household, occupation, or random) on which interactions occur. Another essential attribute is the period of infectivity, modelled with a gamma distribution.

For DeepABM^6^, most simulation parameters follow the values referenced in its CPU-driven predecessor, OpenABM^23^. For the second and third components, age is stratified into nine groups: [0–10, 11–20, 21–30, 31–40, 41–50, 51–60, 61–70, 71–80, and 80]. The susceptibility scalars, $S_{a_s}$, for the nine age groups are [0.35, 0.69, 1.03, 1.03, 1.03, 1.03, 1.27, 1.52, 1.52], while the scale factors for the infector being asymptomatic $A_{s_i}$ are [0.0, 0.33, 0.05, 0.05, 0.72, 1.0, 0.0, 0.0, 0.0, 0.0, 0.0]. The scale factors for interaction duration, $B_n$, are 2, 1, and 0.25 for household, occupation, and random networks, respectively. Regarding the gamma distribution accounting for infection duration, $\mu _i, \sigma _i^2$ are 5.5 and 2.14, respectively. Agent distributions (static parameters) and interactions (attributes of interaction networks) are parameterized using census data.

We considered five uncertain parameters for GSA, described in Table 2. While the first parameter directly corresponds to *R* in the model, and thus did not undergo any transformations, the other four parameters followed a simple transformation from the corresponding parameters in Eq. (25): multiplication of all values for $S_{a_s}$, $A_{s_i}$, $B_{n}$ and both $\mu _i, \sigma _i^2$ in $f_{\Gamma }(u;\mu _i, \sigma _i^2)$ by respective general scale-factors (Table 2). In particular, the entire tensors $S_{a_s}$ and $A_{s_i}$ used in DeepABM-COVID were multiplied by $S_{F}$ and $A_{F}$, respectively. The mean range for *R* was taken from Park et al.^24^. The scalar range for $\mu , \sigma ^2$ in gamma distribution was considered to be close to published estimates^25^. For $S_{F}$, $A_{F}$ and $B_{F}$, the ranges were selected to give a mean of one for corresponding uniform distributions. Using low-discrepancy sequences and the Saltelli sampling method, we generated $N =7168$ samples yielding $\mathcal {X} \in \mathbb {R}^{7168 \times 5}$ (corresponding to 1024 distinct samples used in the Saltelli sampling algorithm). For each input matrix row, we ran 20 repetitions, each with the default 180 time steps and 100,000 agents. Networks and agent identities (static model components) were initialized once prior to all simulations. All model runs were performed on GPUs, leveraging the computational capability of SURF (Samenwerkende Universitaire RekenFaciliteiten) research services and the Dutch National Supercomputer Snellius.

**Table 2:** Details of the uncertain input parameters for the DeepABM-COVID model.

Variable	Distribution	Description
*R*	$\sim \mathcal {U}(1.9, 6.5)$	Multiplicative scalar for overall infection rate
$S_{F}$	$\sim \mathcal {U}(0.5, 1.5)$	General multiplicative scalar for susceptibility
$A_{F}$	$\sim \mathcal {U}(0.5, 1.5)$	General multiplicative scalar for an infector being asymptomatic
$B_{F}$	$\sim \mathcal {U}(0.7, 1.3)$	General multiplicative scalar for interaction duration
$\Gamma _{F}$	$\sim \mathcal {U}(0.7, 1.3)$	General multiplicative scalar for $\mu , \sigma ^2$ in gamma distribution

For conventional GSA, the following five responses were considered in terms of the number of individuals described by a particular state at each time step: “susceptible”, “hospitalized”, “dead”, “recovered”, and “active”. Sobol’ indices were calculated at multiple time steps for each response and averaged over 20 repetitions. For the proposed GSA framework, the same five solutions used for the conventional GSA were concatenated into a single vector, resulting in an output matrix, $\mathcal {Y} \in \mathbb {R}^{7168 \times 900}$, for each of the 20 runs. Each of the 7168 solutions in each run was reshaped into a square matrix, $\varvec{Y}_i \in \mathbb {R}^{30 \times 30}$. For GDMaps, we examined multiple values for *p* and compared the resulting Sobol’ indices across them. We preserved three diffusion coordinates ($g=3$) and investigated the differences between parsimoniously selected diffusion coordinates and those corresponding to the three largest non-trivial eigenvalues (non-parsimonious implementation). We explored four distinct values for the maximum polynomial degree of $s_{\max }$ in constructing the total-degree PCE basis and compared the respective validation errors and how the resulting first- and total-order sensitivity indices changed with larger $s_{\max }$.

#### Agent-based model of poverty trap formation

The last model, used to study the application of the proposed framework on different levels of output, was a multi-scalar ABM of poverty trap formation, proposed by Dupont in^7^. This model facilitates the exploration of how not only individual-level properties but also community-level information influence the emergence of poverty traps. For this purpose, Dupont introduced joint ventures of investing in risky projects that can be undertaken by agents in the same community by forming self-financing groups. As the name suggests, if a risky project does not reach a certain threshold of investment, participating agents lose the amount they invested. At the individual level, agents perform portfolio optimisation and intertemporal optimisation of consumption, which influence one another. Consequently, the model enables explicit analysis at three distinct levels: individual (micro), community (meso), and population (macro), aligning with different temporal scales.

Leveraging the explicit multi-levelness of his model, Dupont performed the GSA approach proposed in this thesis to study how parametric sensitivities change at different levels. In doing so, he utilized two setups with different graph structures—i.e., one with a Holme-Kim graph and another one with a social distance attachment graph. These graphs resulted in different numbers of communities, which were identified using the label propagation algorithm. In the first setup with the Holme-Kim network, six uncertain input parameters summarised in Table 3 were considered. The second setup with the social distance attachment graph involved the examination of parametric sensitivities of only three parameters (*COST*, $\beta$, and $\ell$) with the same distributions as in Table 3, while $G_{\text {upper}}$, $\sigma _w$, and $\lambda$ were fixed to 2, 0.08, and 14, respectively.

**Table 3:** Details of the uncertain input parameters for the poverty trap formation ABM.^7^.

Variable	Distribution	Description
*COST*	$\sim \mathcal {U}(0.01, 2)$	Minimum project investment to avoid failure
$\beta$	$\sim \mathcal {U}(0.7, 0.8)$	Parameter for choosing consumption amount
$\ell$	$\sim \mathcal {U}(0.30, 0.45)$	Probability of project loss
$G_{\text {upper}}$	$\sim \mathcal {U}(1.7, 2.3)$	Upper bound of uniform distribution for project gain
$\sigma _w$	$\sim \mathcal {U}(0.01, 0.15)$	Standard deviation of initial wealth distribution
$\lambda$	$\sim \mathcal {U}(8, 20)$	Expected rate of occurrences for Poisson-distributed portfolio update times

We following the setup with the six-dimensional parameter space used by Dupont^7^, extending the performed GSA by considering additional Grasmannian dimensions, *p*, for micro and meso levels to investigate how sensitivities change for these levels with varying *p*, and the maximum polynomial degrees of $s_{\max } \in \{3, 6, 9, 12\}$ to study the error convergence. We used the same model response data simulated by Dupont with $N=8192$ samples ($\mathcal {X} \in \mathbb {R}^{8192 \times 6}$) and 10 repetitions with fixed random seeds. At the GDMaps step, we preserved $g=3$ parsimoniously selected diffusion coordinates.

The level-based specifications of the data used for each of the 10 repetitions and GSA setups are as follows. At the micro level, the output consisted of time-series data of 50 discrete time points for 1250 agents for each of $N=8192$ parameter combinations. For each of the $N=8192$ solutions, the output was reshaped into a square matrix, $\varvec{Y}_i \in \mathbb {R}^{250 \times 250}$. For GDMaps, we investigated two Grassmannian dimensions of $p\in \{30, 70\}$. At the meso level, the trajectories of wealth for individual communities were computed by calculating the average wealth of agents within the same community at each time step. Following Dupont^7^, the number of communities in the Holme-Kim graph was 267, eight random duplicates of community-level trajectories were generated, and the final six time steps were excluded. This adjustment ensured that the reshaped matrices, corresponding to each of the 8192 model evaluations, remained square. Consequently, the resulting matrices representing the outputs at the community level had the shape of (110, 110). For GDMaps, we examined two Grassmannian dimensions, $p\in \{20, 70\}$. At the macro level, a single wealth trajectory for each of the 8192 solutions was computed as a population average. After removing the last time step, each of 8192 solutions (49-dimensional vectors) was reshaped into a square matrix, $\varvec{Y}_i \in \mathbb {R}^{7 \times 7}$. At this level, we did not explore additional Grassmannian dimensions given a relatively low number of columns in the reshaped matrices and kept $p=4$ as in the analysis done by Dupont^7^. For all three levels, we used the following maximum polynomial degrees: $s_{\max } \in \{3,6,9,12\}$.

### Supplementary Information

Supplementary Information.

## Data Availability

The output data from the DeepABM-COVID simulations is available at https://figshare.com/articles/dataset/output_data_zip/22216921, while the data from the poverty trap formation ABM can be found at https://figshare.com/articles/dataset/ABM_output/24517021.

## Code availability

The code employed to generate data for evaluating the methodology proposed in this research can be accessed at https://github.com/bazvalya/Rethinking_GSA.

## References

[1] Edeling, W., Arabnejad, H., Sinclair, R., Suleimenova, D., Gopalakrishnan, K., Bosakand, B., Groenand, D., Mahmood, I., Crommelin, D., & Coveney, P.V. The impact of uncertainty on predictions of the covidsim epidemiological code. *Nat. Comput. Sci.* **1**, 128–135. 10.1038/s43588-021-00028-9 (2021). 10.1038/s43588-021-00028-9 38217226

[2] Ligmann-Zielinska A, Sun L (2010). Applying time-dependent variance-based global sensitivity analysis to represent the dynamics of an agent-based model of land use change. Int. J. Geogr. Inf. Sci..

[3] Campbell K, McKay MD, Williams BJ (2006). Sensitivity analysis when model outputs are functions. Reliab. Eng. Syst. Saf..

[4] Ligmann-Zielinska, A., Siebers, P.-O., Magliocca, N., Parker, D.C., Grimm, V., Du, J., Cenek, M., Radchuk, V., Arbab, N.N., & Li, S., *et al.* ‘one size does not fit all’: a roadmap of purpose-driven mixed-method pathways for sensitivity analysis of agent-based models. *J. Artif. Soc. Soc. Simul.* **23**(1) (2020).

[5] Kontolati, K., Loukrezis, D., Santos, K.R., Giovanis, D.G., & Shields, M.D. Manifold learning-based polynomial chaos expansions for high-dimensional surrogate models. Int. J. Uncert. Quantif. **12**(4). 10.1615/Int.J.UncertaintyQuantification.2022039936 (2022).

[6] Chopra, A., Gel, E., Subramanian, J., Krishnamurthy, B., Romero-Brufau, S., Pasupathy, K. S., Kingsley, T. C., & Raskar, R. Deepabm: Scalable, efficient and differentiable agent-based simulations via graph neural networks. 10.48550/ARXIV.2110.04421 (2021).

[7] Dupont, C. Complex Systems Analysis of Multi-Level Poverty Traps. Master’s thesis, University of Amsterdam (2023).

[8] Dsilva CJ, Talmon R, Coifman RR, Kevrekidis IG (2018). Parsimonious representation of nonlinear dynamical systems through manifold learning: A chemotaxis case study. Appl. Comput. Harmon. Anal..

[9] Pianosi, F., & Wagener, T.: A simple and efficient method for global sensitivity analysis based on cumulative distribution functions. *Environ. Model. Softw.* **67**, 1–11 (2015). 10.1016/j.envsoft.2015.01.004

[10] Borgonovo E, Plischke E (2016). Sensitivity analysis: A review of recent advances. Eur. J. Oper. Res..

[11] Liu Q, Homma T (2009). A new computational method of a moment-independent uncertainty importance measure. Reliab. Eng. Syst. Saf..

[12] Bazyleva V, Garibay VM, Roy D, Mikyška J, Mulatier C, Paszynski M, Krzhizhanovskaya VV, Dongarra JJ, Sloot PMA (2023). Global sensitivity analysis using polynomial chaos expansion on the grassmann manifold. Computational Science - ICCS 2023.

[13] Smith, R.C. Uncertainty Quantification: Theory, Implementation, and Applications vol. 12, pp. 321–344 (Siam, 2013)

[14] Saltelli A (2002). Making best use of model evaluations to compute sensitivity indices. Comput. Phys. Commun..

[15] Xiu D, Karniadakis GE (2002). The wiener-askey polynomial chaos for stochastic differential equations. SIAM J. Sci. Comput..

[16] Sudret B (2008). Global sensitivity analysis using polynomial chaos expansions. Reliab. Eng. Syst. Saf..

[17] Dos Santos KR, Giovanis DG, Shields MD (2022). Grassmannian diffusion maps-based dimension reduction and classification for high-dimensional data. SIAM J. Sci. Comput..

[18] Hosder, S., Walters, R., & Balch, M. Efficient sampling for non-intrusive polynomial chaos applications with multiple uncertain input variables. In *48th AIAA/ASME/ASCE/AHS/ASC Structures, Structural Dynamics, and Materials Conference*, 1939 (2007).

[19] Fajraoui N, Marelli S, Sudret B (2017). Sequential design of experiment for sparse polynomial chaos expansions. SIAM/ASA J. Uncert. Quantif..

[20] Blatman G, Sudret B (2011). Adaptive sparse polynomial chaos expansion based on least angle regression. J. Comput. Phys..

[21] Efron, B., Hastie, T., Johnstone, I., & Tibshirani, R. Least angle regression (2004).

[22] Lüthen N, Marelli S, Sudret B (2021). Sparse polynomial chaos expansions: Literature survey and benchmark. SIAM/ASA J. Uncert. Quant..

[23] Abueg, M., Hinch, R., Wu, N., Liu, L., Probert, W., Wu, A., Eastham, P., Shafi, Y., Rosencrantz, M., Dikovsky, M., Cheng, Z., Nurtay, A., Abeler-Dörner, L., Bonsall, D., McConnell, M.V., O’Banion, S., & Fraser, C. Modeling the combined effect of digital exposure notification and non-pharmaceutical interventions on the covid-19 epidemic in washington state. medRxiv. 10.1101/2020.08.29.20184135 (2020).

[24] Park M, Cook AR, Lim JT, Sun Y, Dickens BL (2020). A systematic review of covid-19 epidemiology based on current evidence. J. Clin. Med..

[25] Ganyani T, Kremer C, Chen D, Torneri A, Faes C, Wallinga J, Hens N (2020). Estimating the generation interval for coronavirus disease (covid-19) based on symptom onset data, March 2020. Eurosurveillance.

