# Supplementary material for: Trajectory-based global sensitivity analysis in multiscale models
Source: Sci Rep. 2024 Jun 17;14:13902. doi: 10.1038/s41598-024-64331-x (PMC11183117; doi:10.1038/s41598-024-64331-x)
Supplement: Supplementary file 1 — Supplementary Information. [file 41598_2024_64331_MOESM1_ESM.pdf]

Supplementary Information
Trajectory-based Global Sensitivity Analysis in Multiscale Models

Valentina Bazyleva[1*], Victoria M. Garibay[1*] and Debraj Roy[1]

[1]Faculty of Science, Informatics Institute, University of Amsterdam, Science Park 904, Amsterdam, 1098 XH, North Holland, Netherlands.

*Corresponding author(s). E-mail(s): bazvalya@gmail.com; V.M.Garibay@uva.nl;
Contributing authors: D.Roy@uva.nl;

1 Introduction

The supplementary information provides a discussion on some challenges of the conventional global sensitivity analysis (GSA) methods as well as details of theoretical foundations for GSA on a propagator by considering an analogy that can offer us an important insight into how time-dependent GSA can be performed to generalize temporal dynamics of agent-based models (ABMs) at multiple levels of analysis: the Koopman operator theory and dynamic mode decomposition. This document also includes an intuitive explanation of the geometric concepts relevant for understanding the Grassmann manifold and additional findings that offer further insights into the results of applying the proposed GSA method to the three models as discussed in the main text.

2 Limitations of Standard Global Sensitivity Analysis Approaches for Agent-Based Models

GSA belongs to the class of methods that are closely associated with the field of uncertainty quantification (UQ) and examines how robust (sensitive) a model is to specific conditions by estimating the relative effect of individual parameters on model responses [1]. Contrary to local sensitivity analysis, which probes the immediate vicinity of sample input values and assesses the impact of a minor alteration in the input while keeping all other inputs unchanged, GSA evaluates the response variability across all inputs simultaneously [2–4].

Research communities that utilize ABMs have increasingly incorporated GSA for model verification—i.e., inspecting whether the model implementation matches the intended design—and to identify critical factors and errors, streamline model complexity, and enhance understanding of model dynamics and parameter interactions [3–8]. The growing use of GSA signifies ABMs' evolving maturity, shifting from initial, unsystematic applications to adopting structured sensitivity analyses, including the One-at-a-time (OAT) method, and progressing towards acknowledging OAT's limitations and the necessity for comprehensive GSA approaches such as GSA [9].

Variance-based and density-based GSA methods are prominent in ABMs. In variance-based GSA, also known as Sobol' method, the variance of the model response is decomposed, and the sensitivity of uncertain parameters is estimated as the fraction of output variance corresponding to the change in that input alone or in combination with other inputs [10]. The primary reasons for using variance-based methods are that it is applicable independently of the input–output relationships (e.g., linear or non-linear), is user-friendly, and offers straightforward implementation and interpretation [9, 11, 12].

In density-based GSA, cumulative distribution functions of model responses are initialized to estimate their sensitivity to uncertain input parameters of the model [13]. With a recent development called the PAWN index, the density-based approach complements variance-based GSA and addresses the latter's significant limitation. This limitation arises from the assumption at the core of the variance-based method: the second-order moment (i.e., variance) is sufficient to describe response distributions [14]. This assumption can fall short in characterizing non-normal distributions, such as multi-modal or highly-skewed ones, which are frequently observed in complex systems [15, 16].

Standard GSA practices in ABMs, focusing on "final snapshot" sensitivity indices, fail to account for the dynamic nature of ABMs, overlooking the temporal variation in parameter sensitivities [17, 18]. Time-dependent GSA methods (i.e., the time-dependent variance-based approaches) allow for exploring these dynamics, offering a deeper understanding of process-oriented models like ABMs [18, 19].

ABMs, characterized by their rich temporal dynamics and ability to model nonlinear dynamics and emergent system-level patterns such as feedback loops, tipping points, and phase transitions, necessitate a more nuanced sensitivity analysis approach. Time-dependent GSA, as advocated by Ligmann-Zielinska & Sun [18], enhances understanding of changing parametric sensitivities. Nevertheless, such an approach still has crucial limitations related to the first characteristic of ABMs. First, the outcome of the snapshot-based GSA approach can be challenging to interpret when model responses exhibit non-linear behavior like oscillations, transient periods, or tipping points, as the resulting sensitivity indices would oscillate or appear chaotic. Furthermore, the resulting time series of sensitivities would change based on the time points a researcher decides to use for the analysis. Second, time-varying GSA fails to capture any dynamics underlying a model by providing information on what parameters shift response curves up and down, widen or narrow peaks, or move them left or right [20]. In other words, resulting indices lack information generalizing the history of the process.

Moreover, ABMs generate outputs across multiple levels - i.e., micro, meso, and macro, as presented in Figure 1, each providing distinct insights into system behavior. That is, the micro level focuses on individual agents' actions, choices, interactions, and adaptations corresponding to time series data for each of the agents in the system; the meso level provides insights into dynamics within groups or communities with a smaller number of time series outputs; and finally, at the macro level, a single time series captures global behavior and outcomes resulting from collective actions and interactions of all agents. Often, these levels of analysis correspond to varying temporal or spatial scales. Thus, the behaviors of agents in an economy or society occur at different temporal and spatial scales compared to the changes happening to communities or an entire population of agents. Traditional GSA methods, however, predominantly focus on system-level sensitivities, overlooking the granularity offered by micro and meso-level analyses [9]. This limitation underscores the need for a GSA approach that can dissect sensitivities across these varied levels to enrich our understanding of complex system dynamics.

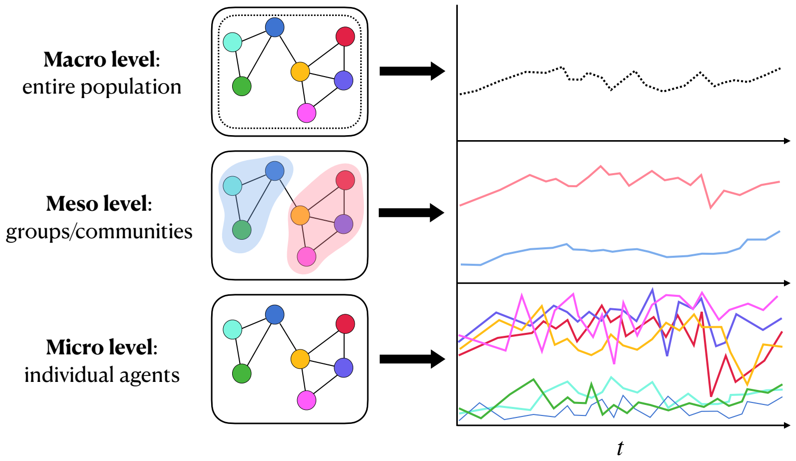

Supplementary Figure 1 Schematic representation of multi-levelness in ABMs as considered in this study.

To harness the full potential of ABMs, the first and, arguably, the most crucial limitation should be addressed [9]. In the field of Reliability Engineering & Risk Assessment, history-aware methods have been

proposed to tackle the problem of insufficiently informative sensitivity indices calculated at multiple discrete time steps [20–24]. This class of methods employs a version of output decomposition or compression (i.e., dimensionality reduction) like PCA. It can also be used to study space-dependent sensitivities [25–28]. The sensitivity indices are either estimated with respect to the basis functions, with the resulting interpretation of parameter effects on the principal components of the model response, or are calculated for the ambient space after the transformation back to the original output, like for applications with spatially-explicit outputs [26].

Although the history-aware methods using PCA from Reliability Engineering & Risk Assessment literature enable a more systematic GSA for time-dependent processes and hence could be useful to study parametric sensitivities of generalized temporal dynamics in ABMs (according to the author's research, none of these techniques have been applied in ABMs as a comprehensive method for time-dependent GSA), PCA as a dimensionality reduction tool can be insufficient to capture the output's intrinsic non-linear geometric structure due to its linearity assumption [29, 30].

Regarding the second limitation, and to the author's knowledge, no method has been proposed to date for estimating parametric sensitivities at agent- and group/community levels in ABMs. The closest idea to examining sensitivities at levels other than model-level may be considered spatial variance-based GSA introduced by Ligmann-Zielinska [31]. While the suggested approach enables independent calculation of sensitivity indices for each defined spatial unit, adapting this method to ABMs without an explicit spatial structure can pose challenges.

3 Agent-based Model as a Propagator

Molecular dynamics simulations provide an insightful analogy to examine inner processes during ABM execution. In particular, the molecular dynamics propagator or transfer operator, referring to a fundamental concept used to describe the evolution of a particle system over time, is essential for understanding the movement and behavior of individual entities in complex systems. This propagator represents the transformation of the initial probability density of states, ρ_0, to a new distribution, ρ_τ, after allowing the system to evolve for a duration, τ, as presented in Figure 2 [32]. Following the mathematical theory of conformation dynamics introduced by Schütte [33], the evolution of a particle ensemble in a time step, τ, can be described by a Markov operator, $\mathcal{P}(\tau)$, as

$$\rho_\tau = \mathcal{P}(\tau)\rho_0. \tag{1}$$

As the molecular dynamics propagator transforms the initial distribution of particles into a new distribution by propagating the evolution of the dynamics over time, ABMs describe the evolution of a system comprised of individual agents interacting with one another and their environment. In ABMs, the state of the system at a given time is determined, at least partially, by the attributes of individual agents, and these attributes evolve throughout the simulation, similar to how positions and velocities of particles in a molecular dynamics simulation change over time. Thus, one can view an ABM simulating the dynamics of a system as a propagator that updates the states of this system at each time step, reflecting agents' decisions and interactions.

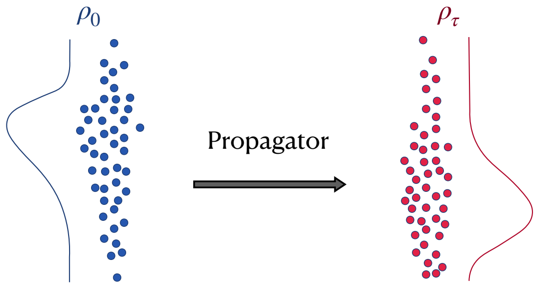

Supplementary Figure 2 A schematic of the molecular dynamics propagator.

When simulating molecular dynamics and reaction kinetics networks, researchers using various modeling techniques (e.g., ABM, ordinary differential equations) are often interested in uncovering metastable dynamics, which are slow processes with long lifetimes. While these slow processes are challenging to simulate directly, as they require long simulation times, they are of great interest in various systems, as they

correspond to rare events that can significantly impact the system's behavior [34]. A general approach to describing slow dynamical processes is by using spectral decomposition of $\mathcal{P}(\tau)$, which can be written as a sum of processes exponentially decaying towards the equilibrium distribution with characteristic time scales, t_i, if the dynamics are at equilibrium [35]:

$$\rho_\tau(\boldsymbol{x}) \approx \sum_i \exp\left(\frac{-\tau}{t_i}\right) \langle \phi_i, \rho_0 \rangle \phi_i(\boldsymbol{x}), \tag{2}$$

where ϕ_i are eigenfunctions. Given the exponential decay of $\exp(-\tau/t_i)$ in the time step, τ, Equation 2 remains an accurate description of the slow processes, which leads to an important realization that we can discretize the high-dimensional system for metastable dynamics, and for this purpose, it is enough to focus on the low-dimensional manifold spanned by eigenfunctions, ϕ_i, of the propagator [34].

Dimensionality reduction techniques are useful for approximating slow-process eigenfunctions like ϕ_i in Equation 2 [35]. One of the most commonly used approaches is principal component analysis (PCA). While PCA can capture the maximum variance within data by projecting it onto a linear subspace, it may not be suitable for extracting the intricate dynamical and relevant non-linear structural information of complex systems like large proteins [36]. Although the linearity assumption of PCA enables an accurate description of local dynamics (i.e., dynamics around one equilibrium point), it can be inadequate for the characterization of the global dynamics of complex systems given that they have multiple equilibria and convoluted energy landscapes [37, 38]. ABMs permit a detailed description of tipping dynamics, resulting in a large number of possible states, and are thus associated with such non-linear and complex landscapes. As a result, non-linear dimensionality reduction techniques, such as diffusion maps (DMaps) [39, 40] or more recently Grassmannian diffusion maps (GDMaps) [29], are needed to obtain a more effective low-dimensional representation of ABM dynamics. With respect to macromolecular systems, GDMaps have been successfully applied to adaptively examine slow-process eigenfunctions and the conformation space [41].

In the context of ABMs, the model response data is often a high-dimensional representation of agent attributes and behaviors over time, especially at the micro and meso analysis levels. In addition, the data matrices obtained from simulating GDMaps are often sparse. However, in high-dimensional sparse data environments, the Euclidean metric used in GDMaps is not appropriate. GDMaps remedies this limitation of conventional DMaps by first projecting the data on the Grassmann manifold, representing the space of all linear subspaces of a fixed dimension within a higher-dimensional space, and then running the diffusion process on the manifold with a non-Euclidean kernel [29]. The resulting latent representation of data on the Grassmann manifold is then used to uncover low-dimensional structures and underlying metastable dynamics. Thus, estimating sensitivity indices with the data on the latent space is analogous to performing GSA on a propagator, which enables systematic generalization of rich temporal dynamics in ABMs.

4 Koopman Operator Theory and Dynamic Mode Decomposition

To further elaborate on the idea of estimating sensitivity indices on a propagator, we turn to the Koopman operator-theoretic perspective and dynamic mode decomposition (DMD). To facilitate this exploration, we adopt equations and a logical framework from the literature addressing this topic [42–46].

In ABMs, we work with discrete-time processes, so let us consider the following discrete-time dynamical system:

$$\boldsymbol{x}_{k+1} = F(\boldsymbol{x}_k), \tag{3}$$

where F is a flow map inducing the dynamics. The Koopman operator theory suggests that the finite-dimensional non-linear behavior of Equation 3 can be converted into an infinite-dimensional linear dynamical system. That is, the Koopman operator, $\mathcal{K} : \mathcal{F} \mapsto \mathcal{F}$, when applied to dynamical systems, operates on observable measurements (i.e., measurement functions), $g \in \mathcal{F}$, defined on the state space of the system [42]. It maps an observable's value at one time to its value at a future time for the discrete-type dynamical system as

$$\mathcal{K}_{\Delta t} g(\boldsymbol{x}_k) = g(F(\boldsymbol{x}_k)) = g(\boldsymbol{x}_{k+1}), \tag{4}$$

where Δt is a discrete time step. From Equation 4, we can obtain the Koopman dynamical system:

$$g(\boldsymbol{x}_{k+1}) = \mathcal{K}_{\Delta t} g(\boldsymbol{x}_k), \tag{5}$$

which is analogous to Equation 3.

We can draw a parallel between the propagator as in Equation 1, describing the evolution of a particle ensemble in time, and the Koopman operator as in Equation 5, capturing the discrete-time evolution of observables in a system without explicitly describing the trajectories of individual state variables. Both operators characterize the system's dynamics, albeit in different contexts (i.e., molecular dynamics versus non-linear systems), and involve understanding how systems evolve over time and can be used to uncover patterns, behaviors, and invariant structures.

DMD is an inherently data-driven method developed by Schmid [43] for extracting coherent spatiotemporal modes from high-dimensional data. Its connection to the Koopman operator was revealed by showing that the dynamic modes acquired through DMD tend to approach the Koopman modes for a specific collection of linear observables [47]. Here, we present the steps of this method that allow us to explain the idea of GSA on a propagator.

The DMD algorithm estimates the linear operator (i.e., propagator matrix), $\boldsymbol{A} \in \mathbb{R}^{n\times n}$, mapping the current system state to the next state as $\boldsymbol{x}_{k+1} = \boldsymbol{A}\boldsymbol{x}_k$, where $\boldsymbol{x}_k \in \mathbb{R}^n$. Note the resemblance between the matrix, $\boldsymbol{A}$, and the Koopman operator, $\mathcal{K}$, in Equation 5. DMD approximates $\boldsymbol{A}$ from the snapshots of states in a dynamical system. For this purpose, it constructs a matrix, $\boldsymbol{X}_1$, consisting of snapshots of simulated measurements, $\boldsymbol{x}_k$, collected at regular time intervals, and its time-delayed version, $\boldsymbol{X}_2$, such that $\boldsymbol{X}_2 \approx \boldsymbol{A}\boldsymbol{X}_1$. The approximation of $\boldsymbol{A}$ can be written as

$$\boldsymbol{A} = \boldsymbol{X}_2 \boldsymbol{X}_1^{+}, \tag{6}$$

where $^+$ denotes the Moore-Penrose inverse (i.e., pseudoinverse) [45, 46]. The pseudoinverse $\boldsymbol{X}_1^+$ can be approximated via the SVD of $\boldsymbol{X}_1$, circumventing the issue of expensive direct eigendecomposition of $\boldsymbol{A}$. The singular value decomposition SVD of $\boldsymbol{X}_1$ reads:

$$\boldsymbol{X}_1 \approx \boldsymbol{U}\boldsymbol{\Sigma}\boldsymbol{V}^{\top}, \tag{7}$$

where $\boldsymbol{\Sigma} \in \mathbb{R}^{r\times r}$ is a diagonal matrix including singular values, $\boldsymbol{U} \in \mathbb{R}^{n\times r}$ and $\boldsymbol{V} \in \mathbb{R}^{m\times r}$ are orthonormal matrices, and r is the reduced rank of the SVD approximation.

We can plug the pseudoinverse of $\boldsymbol{X}_1$ (as in Equation 7) into Equation 6 to obtain the full matrix, $\boldsymbol{A}$, as

$$\boldsymbol{A} \approx \boldsymbol{X}_2 \boldsymbol{V}\boldsymbol{\Sigma}^{-1}\boldsymbol{U}^{\top}, \tag{8}$$

or its approximated version:

$$\widetilde{\boldsymbol{A}} \approx \boldsymbol{U}^{\top}\boldsymbol{X}_2 \boldsymbol{V}\boldsymbol{\Sigma}^{-1}, \tag{9}$$

since, in practice, only the leading r eigendirections of $\boldsymbol{A}$ are of interest [46].

The DMD method can further be used to define the spectral decomposition of $\widetilde{\boldsymbol{A}}$, allowing the reconstruction of the time dynamics of a trajectory (for details, we refer the interested reader to [45, 46]). However, we wish to examine Equations 8 and 9 to understand how DMD connects to applying GSA to a propagator. Matrix, $\boldsymbol{A}$, corresponds to the Koopman operator resembling a molecular dynamics propagator, which, in turn, is reminiscent of how an ABM simulates the dynamics of a system comprised of numerous agents. When we change uncertain parameters, what changes in Equations 8 and 9 are $\boldsymbol{\Sigma}^{-1}$, $\boldsymbol{U}^{\top}$, and $\boldsymbol{V}$, where the last two relate to points on the Grassmann manifold as we will see in the following chapter. Here, changes in the matrix, $\boldsymbol{X}_2$, are inconsequential to the explanation of GSA performed on a propagator. Understanding how changes in the orthonormal matrices also lead to changes in $\boldsymbol{A}$ constitutes the central question of applying GSA on a propagator.

5 Grassmann Manifold: Concepts and Geometric Intuition

The Grassmann manifold, denoted by $\mathcal{G}(p, n)$, is a fundamental concept in differential geometry, representing the set of all p-dimensional linear subspaces in an n-dimensional Euclidean space, $\mathbb{R}^n$. To grasp its implications, especially for applications like DMD and GDMaps, we explore its geometric aspects, focusing on a low dimensional manifold, $\mathcal{G}(1, 3)$, or 1-dimensional linear subspaces (i.e., lines through the origin) in a 3-dimensional Euclidean space, $\mathbb{R}^3$.

Tangent Space

At any point on the Grassmann manifold, the tangent space can be thought of as the 'flat' space that best approximates the manifold locally. Consider $\mathcal{G}(1, 3)$, the space of lines through the origin in $\mathbb{R}^3$.

Each point on this manifold can be visualized as a direction vector in 3D space. The tangent space at any such line is the set of vectors orthogonal to it, representing directions in which the line can 'pivot'. If we imagine a curve on the surface of the Earth, the tangent space at any point on this curve is akin to a piece of paper that touches the globe at that point, offering a local, linear approximation of its surface.

Exponential and Logarithmic Maps

The exponential map transitions from the tangent space back to the manifold, akin to moving from the paper to the globe's surface along the shortest path. Conversely, the logarithmic map projects points from the manifold to the tangent space, like tracing the shortest path from the globe back to the paper:

$$\exp_{\boldsymbol{U}}(\boldsymbol{V}) : T_{\boldsymbol{U}}\mathcal{G} \to \mathcal{G}, \quad \log_{\boldsymbol{U}}(\boldsymbol{V}) : \mathcal{G} \to T_{\boldsymbol{U}}\mathcal{G}, \tag{10}$$

where $\boldsymbol{U}$ and $\boldsymbol{V}$ are points on $\mathcal{G}(1,3)$, and $T_{\boldsymbol{U}}\mathcal{G}$ denotes the tangent space at $\boldsymbol{U}$.

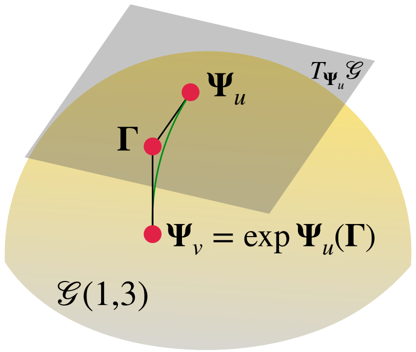

Supplementary Figure 3 Exponential map of a point $\boldsymbol{\Gamma} \in T_{\boldsymbol{\Psi}_u}\mathcal{G}(1,3)$, with two points, $\boldsymbol{U} = \text{span}(\boldsymbol{\Psi}_u)$ and $\boldsymbol{V} = \text{span}(\boldsymbol{\Psi}_v)$.

Geodesic Path

The geodesic path represents the shortest route between two points on the manifold. In $\mathcal{G}(1,3)$, imagine bending a wire to directly connect two dots on a curved surface without leaving the surface. This path is the geodesic. Mathematically, for points $\boldsymbol{U}$ and $\boldsymbol{V}$ on $\mathcal{G}(1,3)$, their geodesic path can be expressed as:

$$\gamma(t) = \exp_{\boldsymbol{U}}(t \log_{\boldsymbol{U}}(\boldsymbol{V})), \quad t \in [0,1], \tag{11}$$

where t is a parameter that varies from 0 to 1, tracing the path from $\boldsymbol{U}$ to $\boldsymbol{V}$.

Distances and Metrics

On $\mathcal{G}(1,3)$, the distance between two lines (subspaces) can be quantified through metrics. A commonly used metric is the geodesic distance, which measures the shortest path between two points on the manifold's surface. For lines in $\mathbb{R}^3$, this distance reflects how 'angled' or 'separate' they are, with parallel lines having zero distance.

Connecting Grassmannian Diffusion Maps to Koopman Operator Theory

Both the GDMaps and the Koopman Operator Theory aim to represent high-dimensional dynamical systems in a lower-dimensional space. The Grassmann manifold's role in GDMaps, where points represent subspaces indicative of the system's states, parallels the Koopman operator's approach of transforming nonlinear dynamics into a linear problem in an infinite-dimensional function space.

The Koopman Operator Theory linearizes nonlinear dynamical systems by operating on observables instead of directly on the state space. Similarly, GDMaps reduce the dimensionality of data nonlinearly, but they begin by linearly projecting high-dimensional data onto the Grassmann manifold through SVD and selecting p modes. This initial linear step is somewhat analogous to finding a linear subspace that best represents the data (or system's dynamics) before applying nonlinear techniques to understand the manifold's geometry.

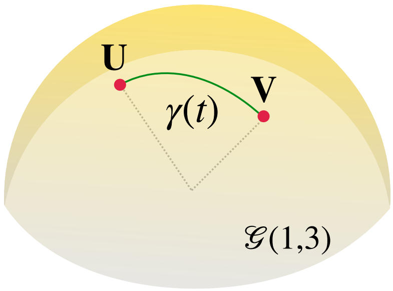

Supplementary Figure 4 Geodesic distance between subspaces $\boldsymbol{U}$ and $\boldsymbol{V}$ of $\mathbb{R}^1$ on $\mathcal{G}(1,3)$.

In GDMaps, as extensively described in the main text, the eigenvalues obtained from the diffusion process on the manifold help in identifying the most significant directions of data variability, which are crucial for understanding the system's intrinsic geometry. Similarly, in Koopman theory, the eigenvalues of the Koopman operator indicate the growth rates, frequencies, and damping rates of dynamic modes, providing insight into the system's fundamental dynamics.

When applying GDMaps to an ABM's output as a time series for varying parameter combinations, we first project this data onto the Grassmann manifold, identifying subspaces that capture the system's essential dynamics. The subsequent nonlinear diffusion process on this manifold could be considered as exploring various paths (geodesics) that connect different system states, analogous to how the Koopman operator connects states over time through its action on observables.

6 Supplementary Results

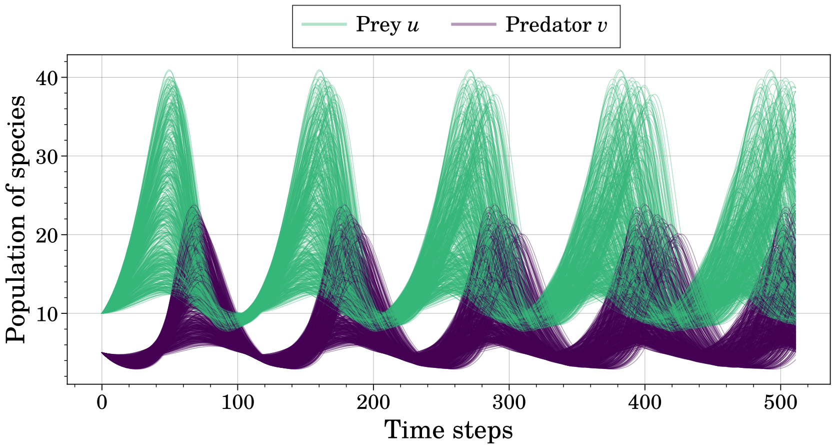

Supplementary Figure 5 Time series of $N = 600$ realisations of the Lotka-Volterra model with two uncertain parameters, $\alpha \sim \mathcal{U}(0.90, 1.05)$ and $\beta \sim \mathcal{U}(0.10, 0.18)$, as inputs randomly sampled from respective distributions. The model outputs for the defined parameter ranges for α and β exhibit oscillatory behavior.

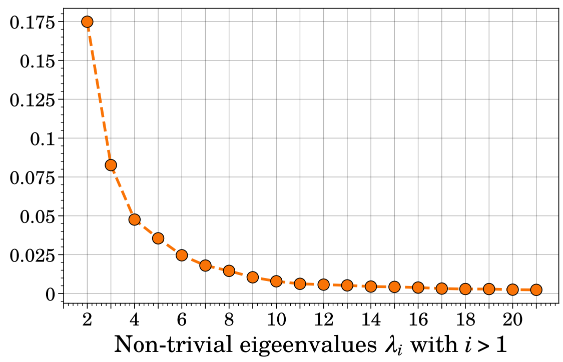

Supplementary Figure 6 Scree-plot displaying eigenvalues obtained from GDMaps applied to the Lotka-Volterra model output..

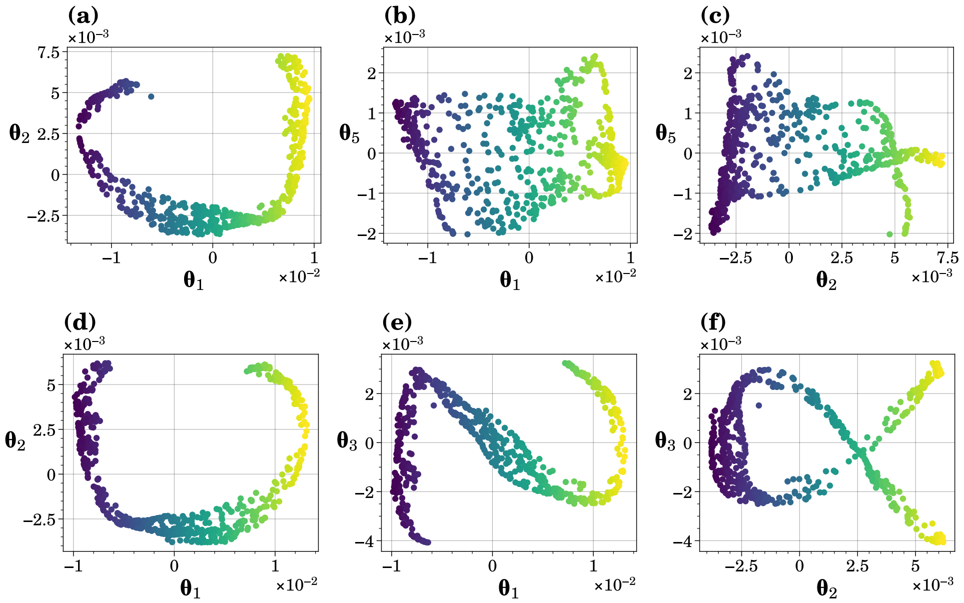

Supplementary Figure 7 2D plots of three diffusion coordinates from GDMaps on the Lotka-Volterra model solutions with two uncertain parameters, α and β (as presented in Table 1 in the Methods section in the main text), as input and $N = 600$ training samples, using the dimension of the Grassmannian $p = 6$. Diffusion coordinates converged to $\vartheta = \{\boldsymbol{\theta}_1, \boldsymbol{\theta}_2, \boldsymbol{\theta}_5\}$ for parsimonious representation (top row). The color maps are defined by $\boldsymbol{\theta}_1$ (a, b) and $\boldsymbol{\theta}_2$ (c).

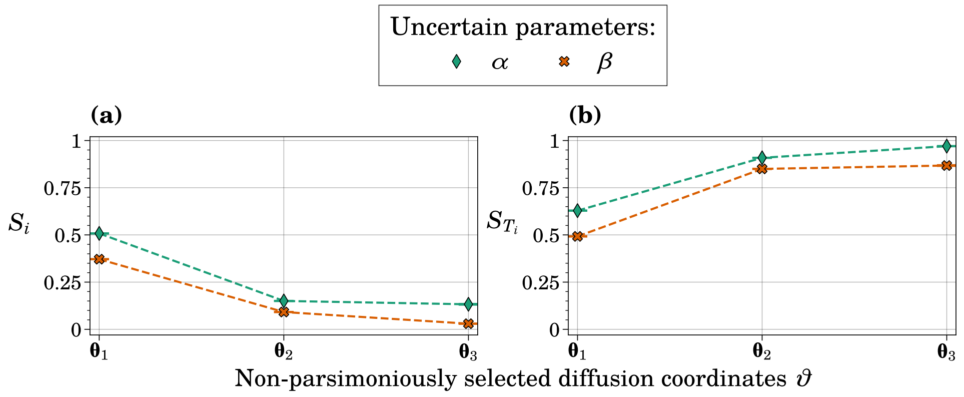

Supplementary Figure 8 Estimates of first- and total-order sensitivity indices, S_i and S_{T_i}, respectively, of α, and β (Table 1 in Methods of in the main text) for two model output measures: number of prey per time step u, and number of predators per time step, v. Error bars indicate variance from 50 resamples. The presented results are for non-parsimoniously selected diffusion coordinates. For GDMaps PCE, Grassmannian dimension of $p = 10$ and maximum polynomial degree of $s_{\max} = 6$ were used.

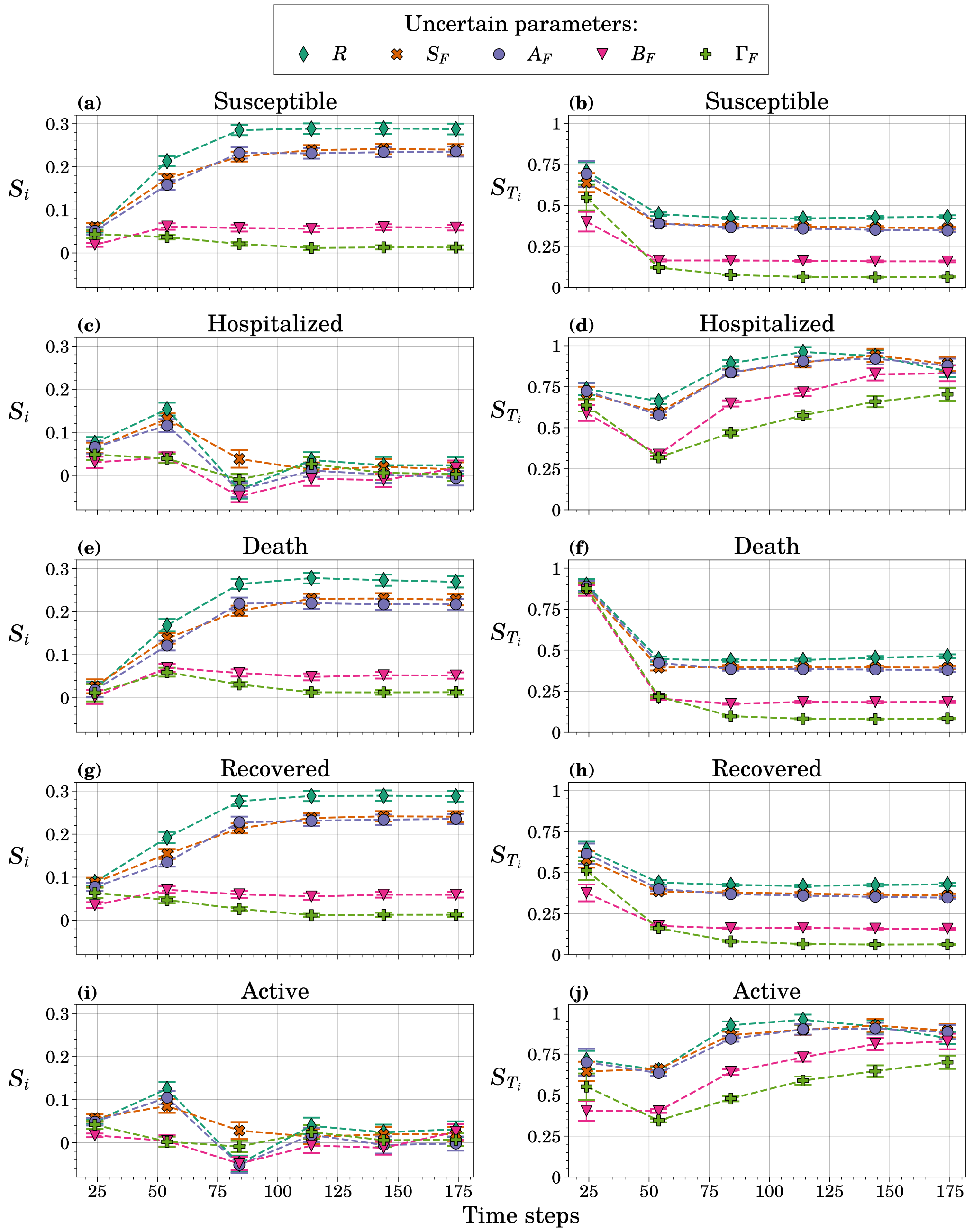

Supplementary Figure 9 Estimates of first- and total-order sensitivity indices for five input parameters (as presented in Table 2 in the Methods section of the main text) across five model outputs using Sobol' indices at six evenly spaced time steps, averaged over 20 runs. Error bars represent 95% bootstrap confidence intervals. Comparable patterns are observed for "susceptible", "death", and "recovered", as well as "hospitalized" and "active" due to their direct relationships. For "hospitalized" and "active", most of the variance in the model output is caused by interaction effects, while "susceptible", "death," and "recovered" show higher main effect indices for R, A_F, and S_F. B_F and Γ_F exhibit the smallest estimates of S_i and S_{T_i} for "susceptible", "death", and "recovered" and of S_{T_i} for "hospitalized" and "active" outputs.

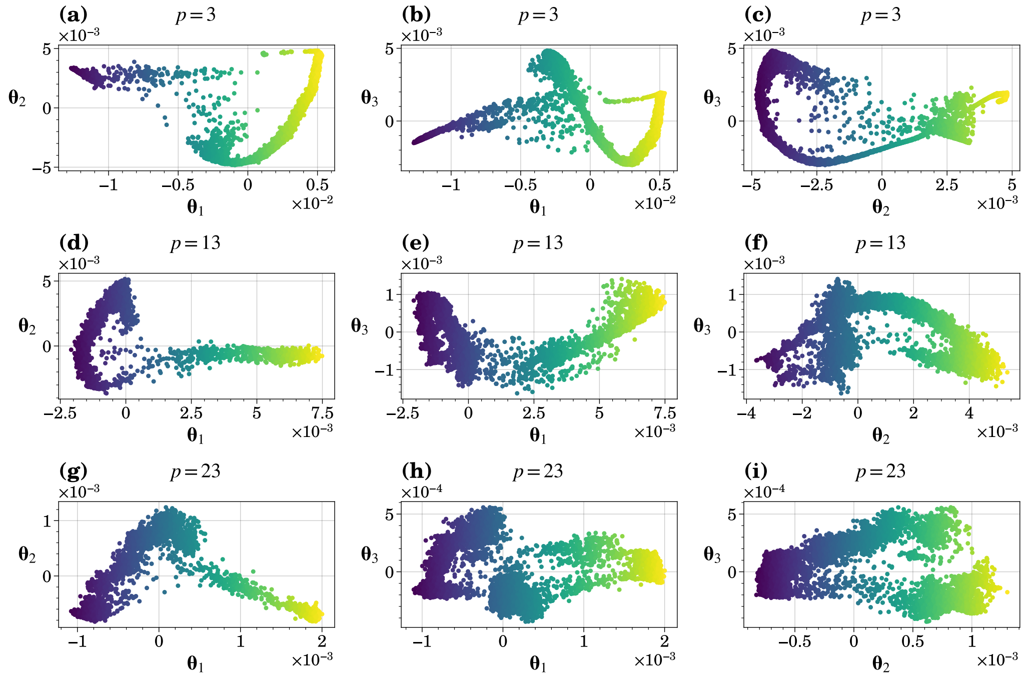

Supplementary Figure 10 2D plots illustrating the first three non-trivial diffusion coordinates (non-parsimonious representation) from GDMaps applied to the DeepABM-COVID model output for run 16, utilizing six distinct Grassmannian dimensions, $p \in \{3, 13, 23\}$. Color maps are determined by the diffusion coordinates on the x-axis. Lower Grassmannian dimensions are associated with slower timescales discovered by GDMaps, as evidenced by the axes' scales.

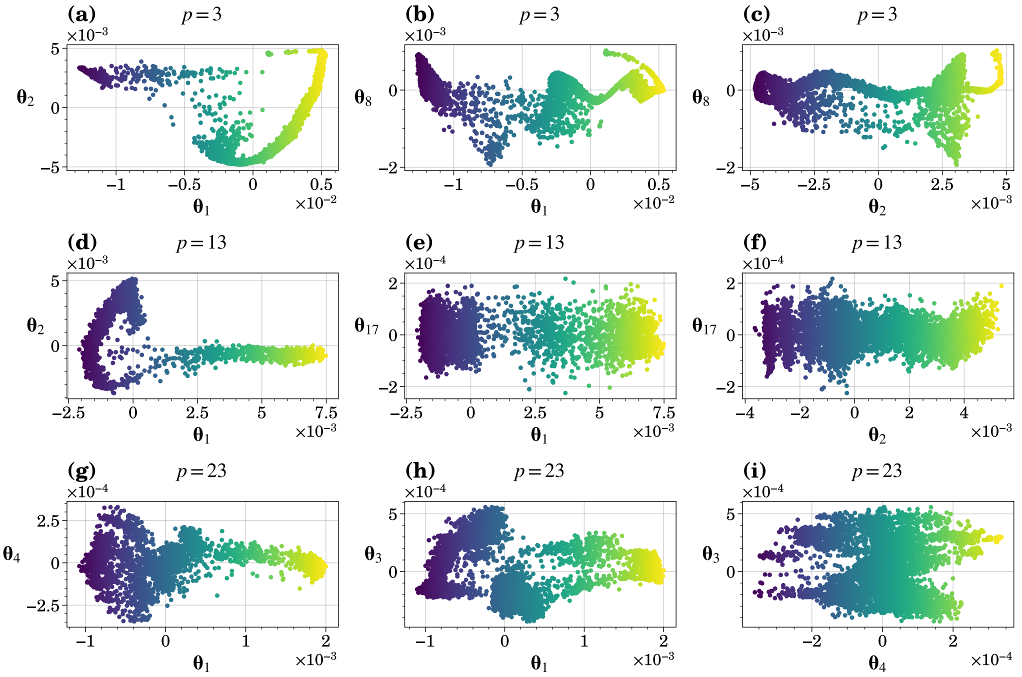

Supplementary Figure 11 2D plots of three diffusion coordinates from GDMaps applied to the DeepABM-COVID model output for run 16, employing a parsimonious representation and six distinct Grassmannian dimensions, $p \in \{3, 13, 23\}$. Color maps are determined by the diffusion coordinates on the x-axis. As the Grassmannian dimension, p, increases, the method more frequently selects diffusion coordinates with lower corresponding eigenvalues (higher subscript values) compared to smaller p values, as evident in the left column.

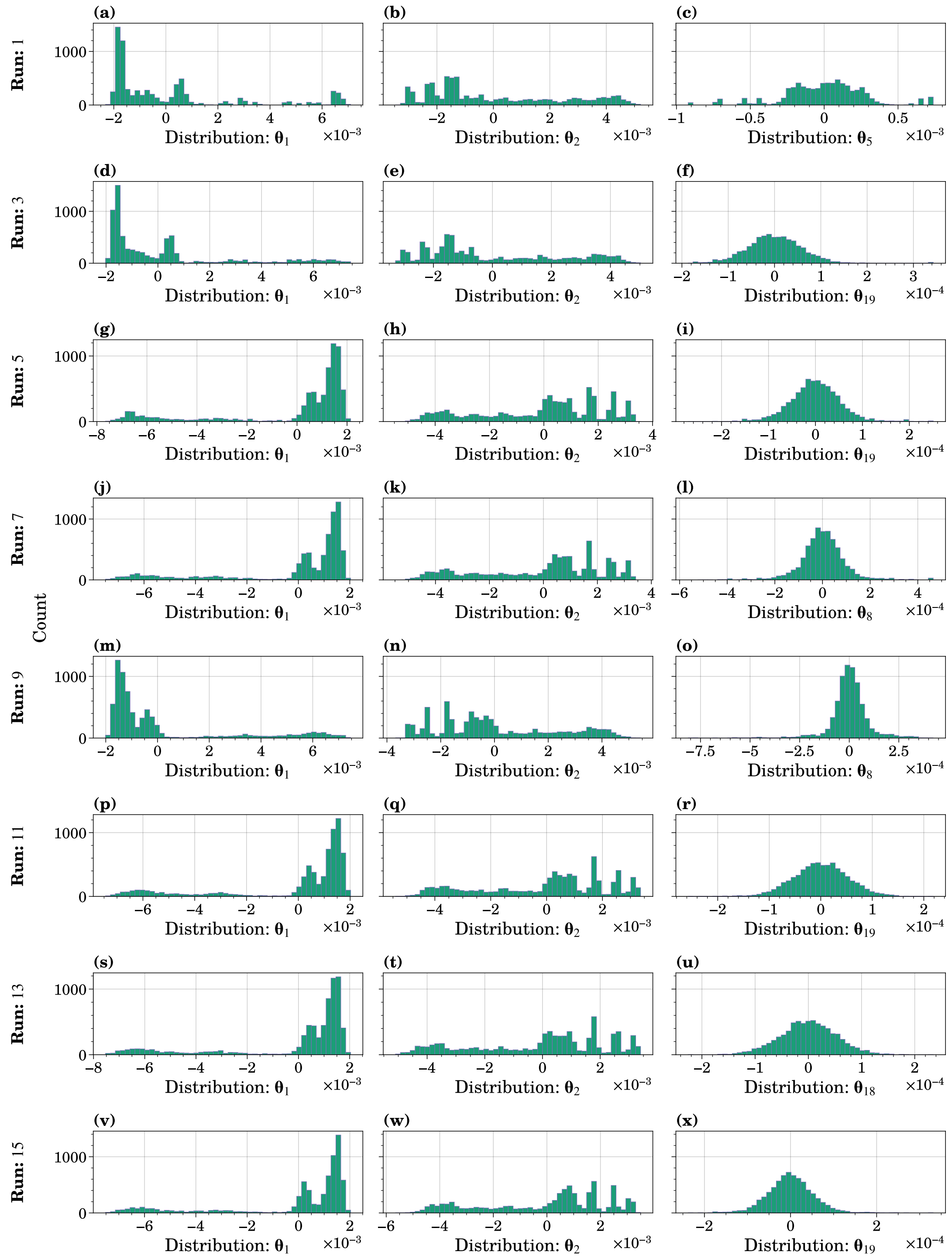

Supplementary Figure 12 Distributions of parsimoniously selected diffusion coordinates for the Grassmannian dimension $p = 13$ obtained from eight distinct runs: $\{1, 3, 5, 7, 9, 11, 13, 15\}$ of the DeepABM-COVID model with resulting output, $\mathcal{Y} \in \mathbb{R}^{7168\times900}$. Higher subscript values correspond to lower non-trivial eigenvalues, λ_i, ordered from high to low. The distributions of diffusion coordinates associated with higher eigenvalues exhibit multi-modal characteristics, while those corresponding to lower eigenvalues display unimodal and even Gaussian-like features.

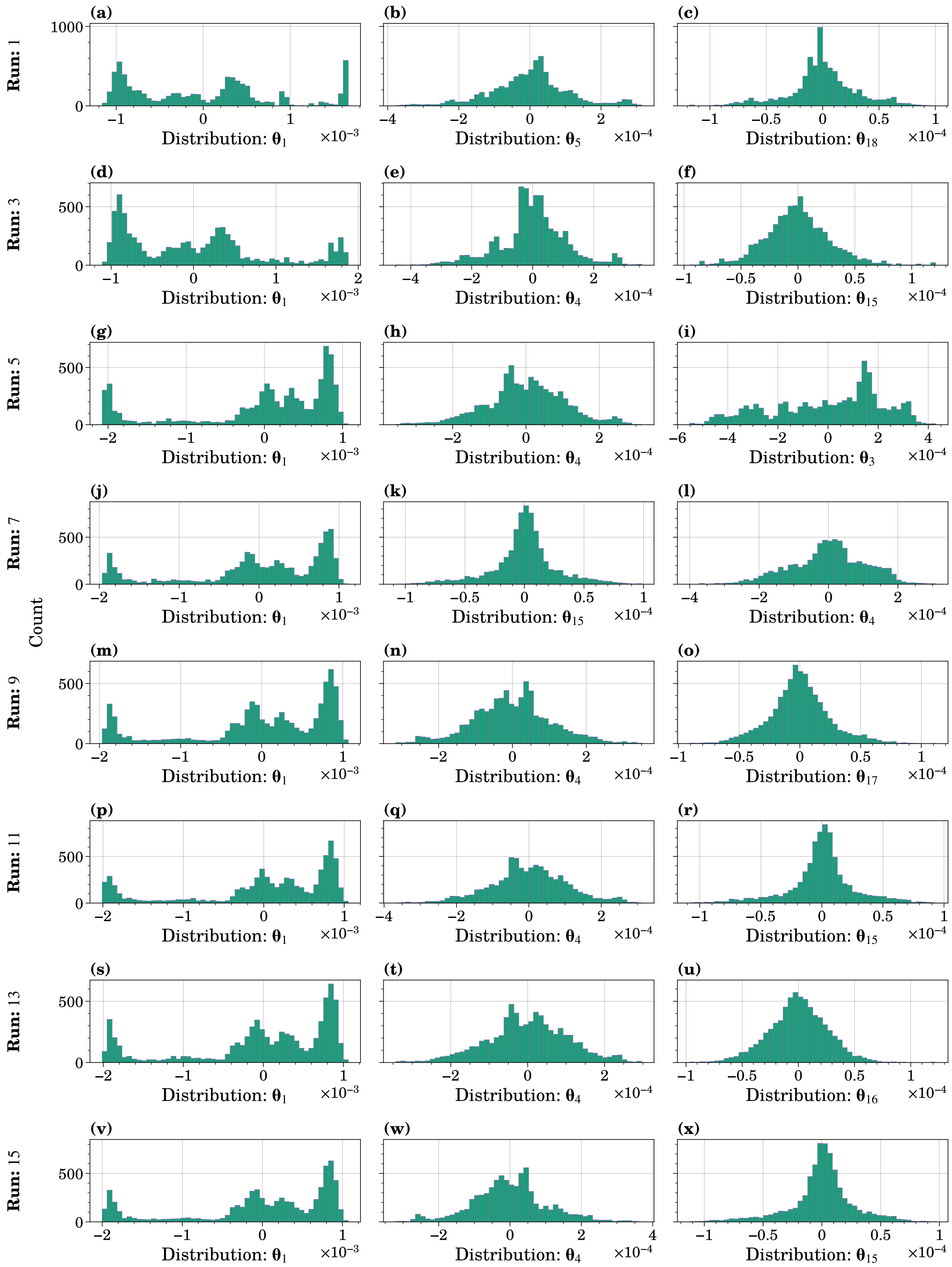

Supplementary Figure 13 Distributions of parsimoniously selected diffusion coordinates for the Grassmannian dimension $p = 23$ obtained from eight distinct runs: $\{1, 3, 5, 7, 9, 11, 13, 15\}$ of the DeepABM-COVID model with resulting output $\mathcal{Y} \in \mathbb{R}^{7168\times 900}$. Higher subscript values correspond to lower non-trivial eigenvalues, λ_i, ordered from high to low. The distributions of diffusion coordinates associated with higher eigenvalues exhibit multi-modal characteristics, while those corresponding to lower eigenvalues display unimodal and even Gaussian-like features.

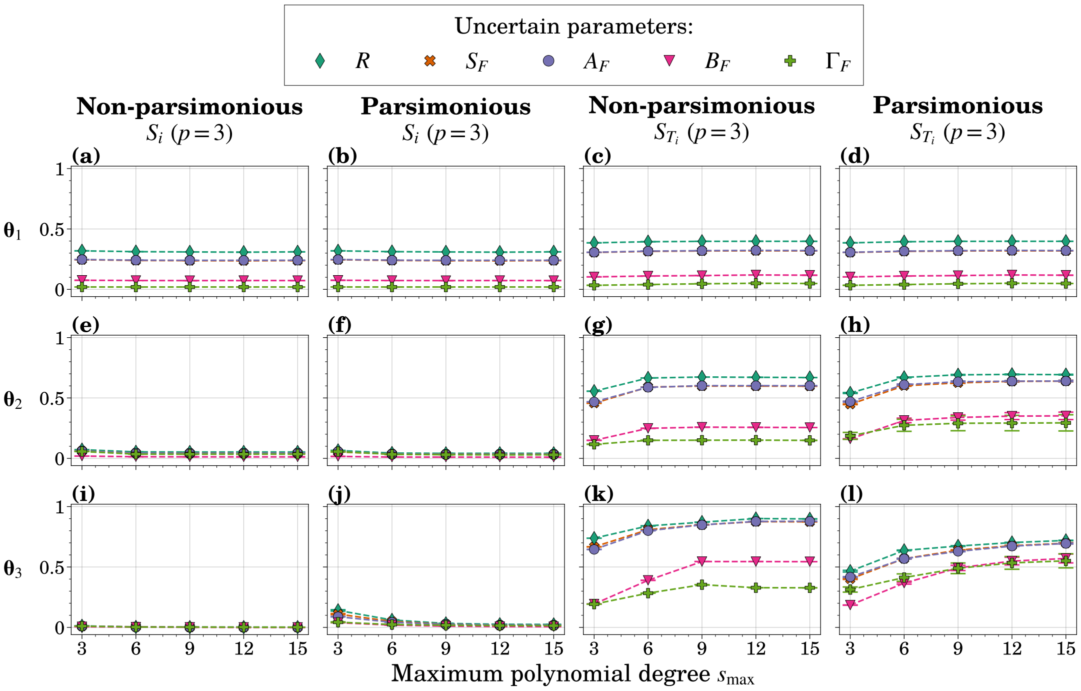

Supplementary Figure 14 DeepABM-COVID: estimates of first- and total-order sensitivity indices (S_i and S_{T_i}) of six uncertain input parameters (see Table 2 Methods section of the main text) for five maximum polynomial degrees, $s_{\max} \in \{3, 6, 9, 12, 15\}$. Sobol' indices were computed from PCE coefficients estimated with LAR on the manifold discovered with GDMaps using the Grassmannian dimension of $p = 3$ and the diffusion coordinates are non-parsimoniously selected. Error bars indicate variance across 10 runs.

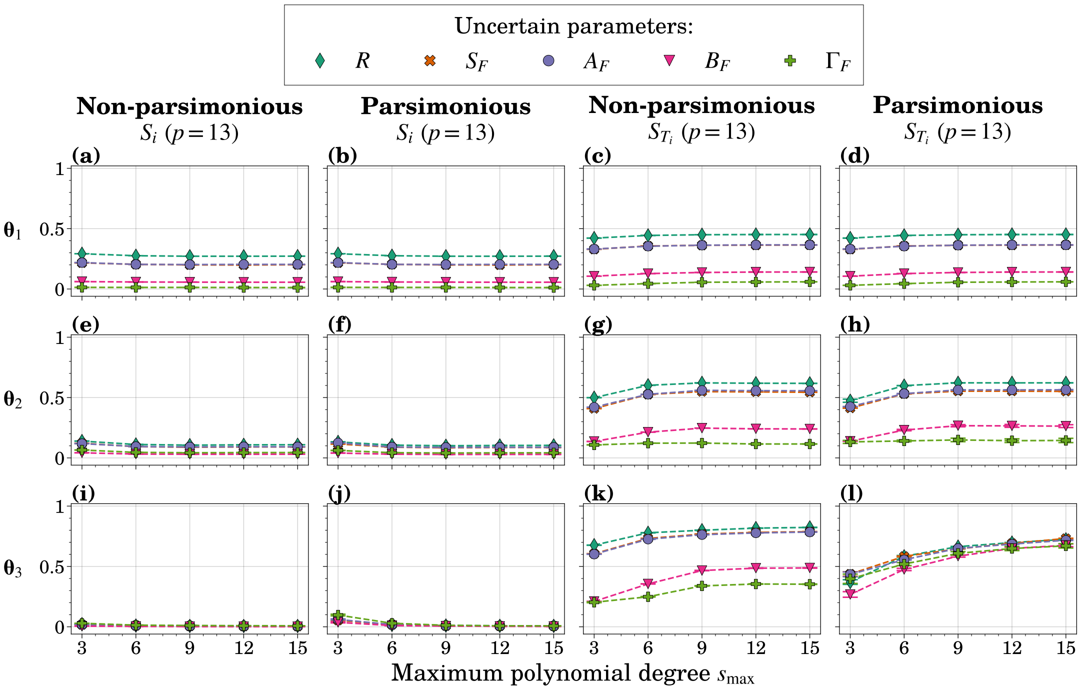

Supplementary Figure 15 DeepABM-COVID: estimates of first- and total-order sensitivity indices (S_i and S_{T_i}) of six uncertain input parameters (see Table 2 Methods section of the main text) for five maximum polynomial degrees, $s_{\max} \in \{3, 6, 9, 12, 15\}$. Sobol' indices were computed from PCE coefficients estimated with LAR on the manifold discovered with GDMaps using the Grassmannian dimension of $p = 13$ the diffusion coordinates are non-parsimoniously selected. Error bars indicate variance across 10 runs.

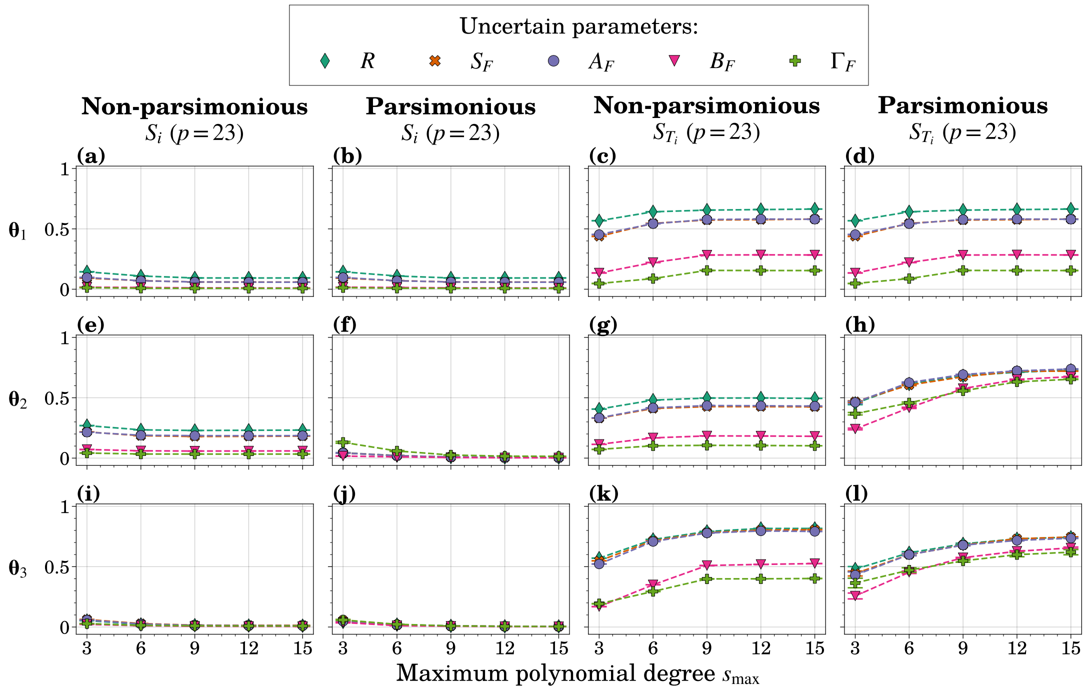

Supplementary Figure 16 DeepABM-COVID: estimates of first- and total-order sensitivity indices (S_i and S_{T_i}) of six uncertain input parameters (see Table 2 Methods section of the main text) for five maximum polynomial degrees, $s_{\max} \in \{3, 6, 9, 12, 15\}$. Sobol' indices were computed from PCE coefficients estimated with LAR on the manifold discovered with GDMaps using the Grassmannian dimension of $p = 23$ the diffusion coordinates are non-parsimoniously selected. Error bars indicate variance across 10 runs.

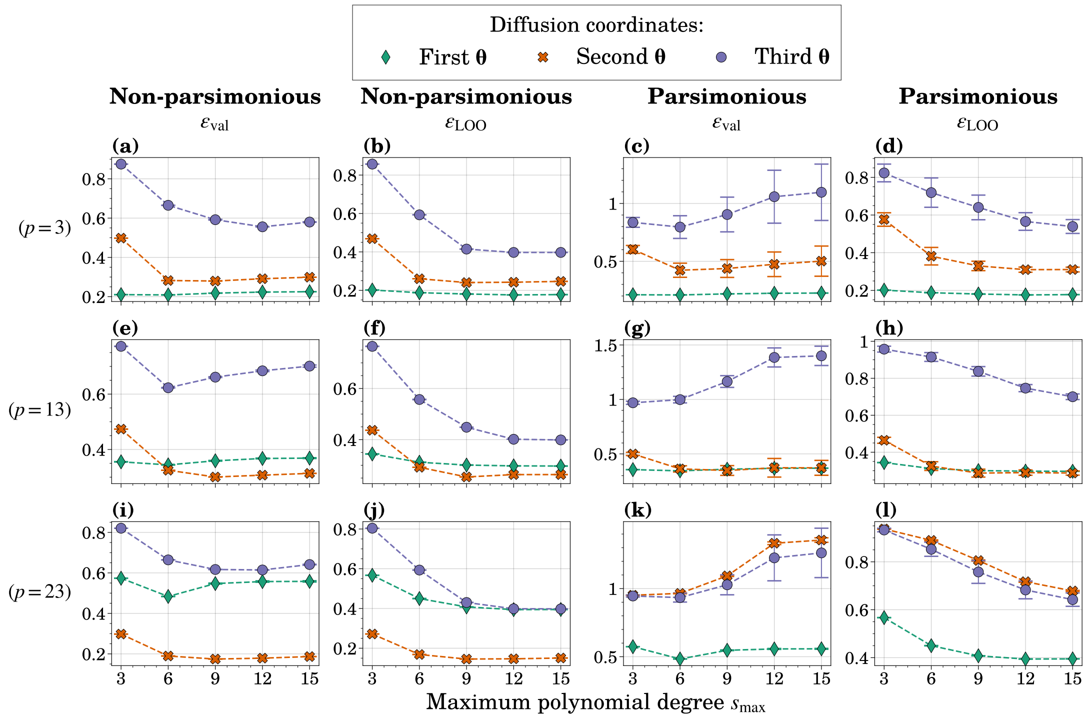

Supplementary Figure 17 DeepABM-COVID: validation and LOO errors (ϵ_{LOO} and ϵ_{val}, respectively) from GDMaps PCE using LAR for the three parsimoniously selected diffusion coordinates and five maximum polynomial degrees, $s_{\max} \in \{3, 6, 9, 12, 15\}$.

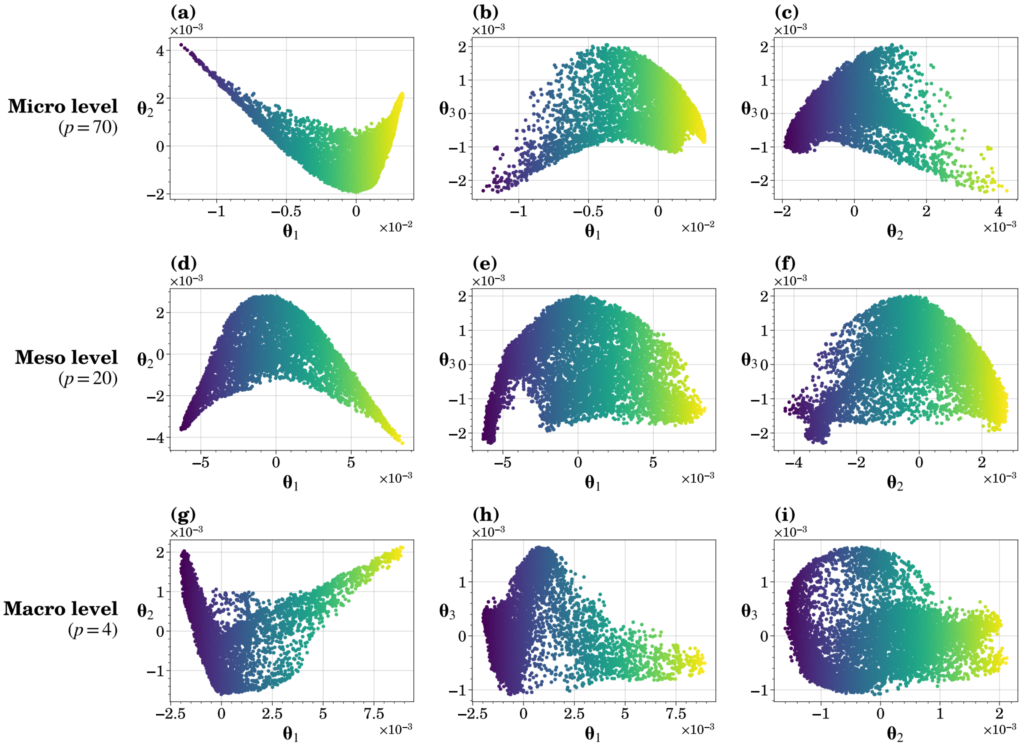

Supplementary Figure 18 2D plots of the first three non-parsimoniously selected diffusion coordinates ϑ obtained from applying GDMaps on the model outputs of the poverty trap formation ABM with three uncertain input parameters at the micro, meso, and macro levels for the chosen manifold dimensions, p. Diffusion coordinates were selected using parsimonious representation. The plots show the resulting diffusion coordinates of only one stochastic repetition (out of 10). Color maps are determined by the diffusion coordinates, $\boldsymbol{\theta}_i$, on the x-axis.

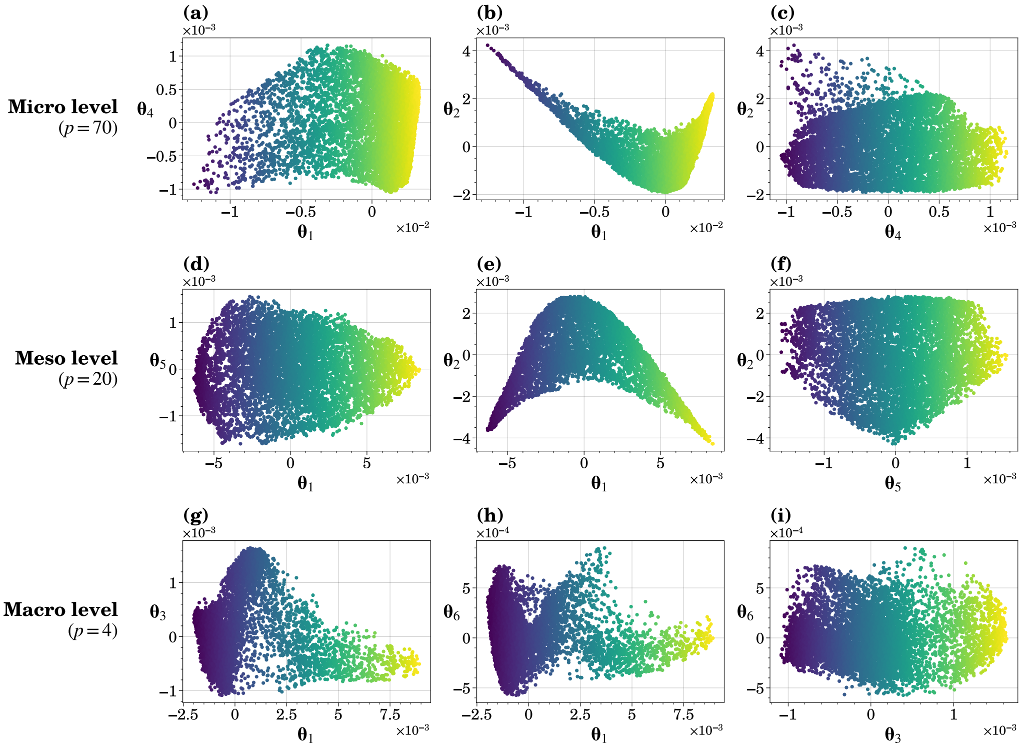

Supplementary Figure 19 2D plots of three parsimoniously selected diffusion coordinates ϑ obtained from applying GDMaps on the model outputs of the poverty trap formation ABM with three uncertain input parameters at the micro, meso, and macro levels for the chosen manifold dimensions, p. The plots show the resulting diffusion coordinates of only one stochastic repetition (out of 10). Color maps are determined by the diffusion coordinates, $\boldsymbol{\theta}_i$, on the x-axis.

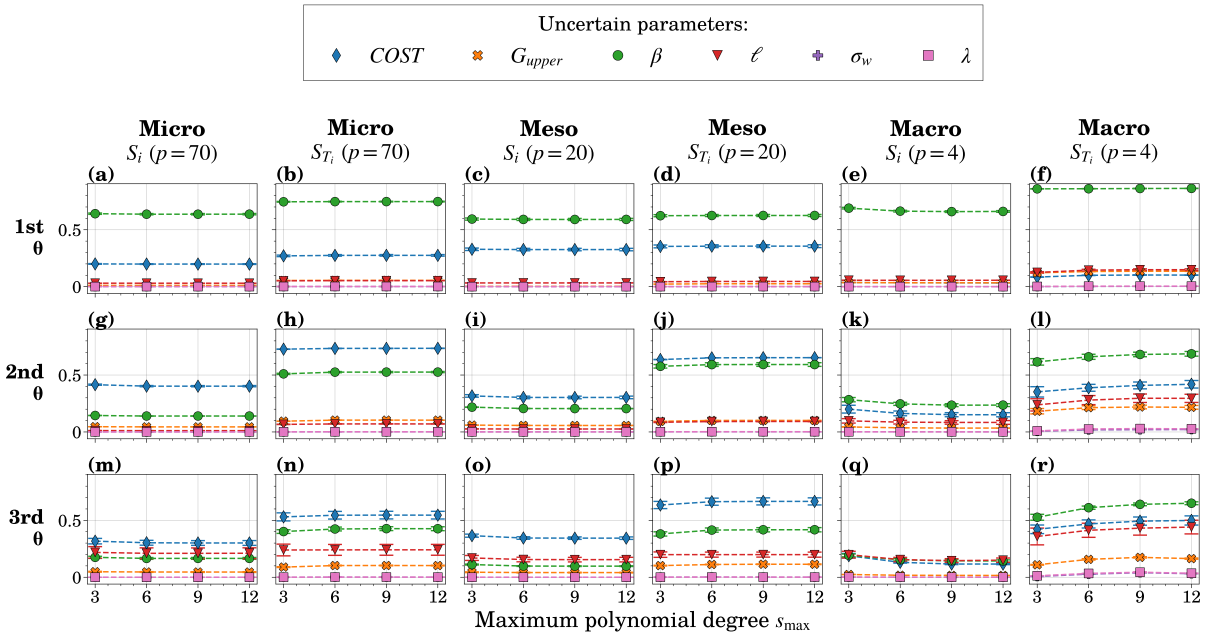

Supplementary Figure 20 Poverty trap formation ABM, micro, meso, and macro levels: estimates of first- and total-order sensitivity indices (S_i and S_{T_i}) of six uncertain input parameters (see Table 3 in Methods section of the main text) for four maximum polynomial degrees, $s_{\max} \in \{3, 6, 9, 12\}$. Sobol' indices were computed from PCE coefficients estimated with LAR on the manifold discovered with GDMaps using two Grassmannian dimensions of $p = 70$ for the micro level, $p = 20$ for the meso level, and $p = 4$ for the macro level. For the selection of diffusion coordinates, non-parsimonious representation was used. Error bars indicate variance across 10 runs.

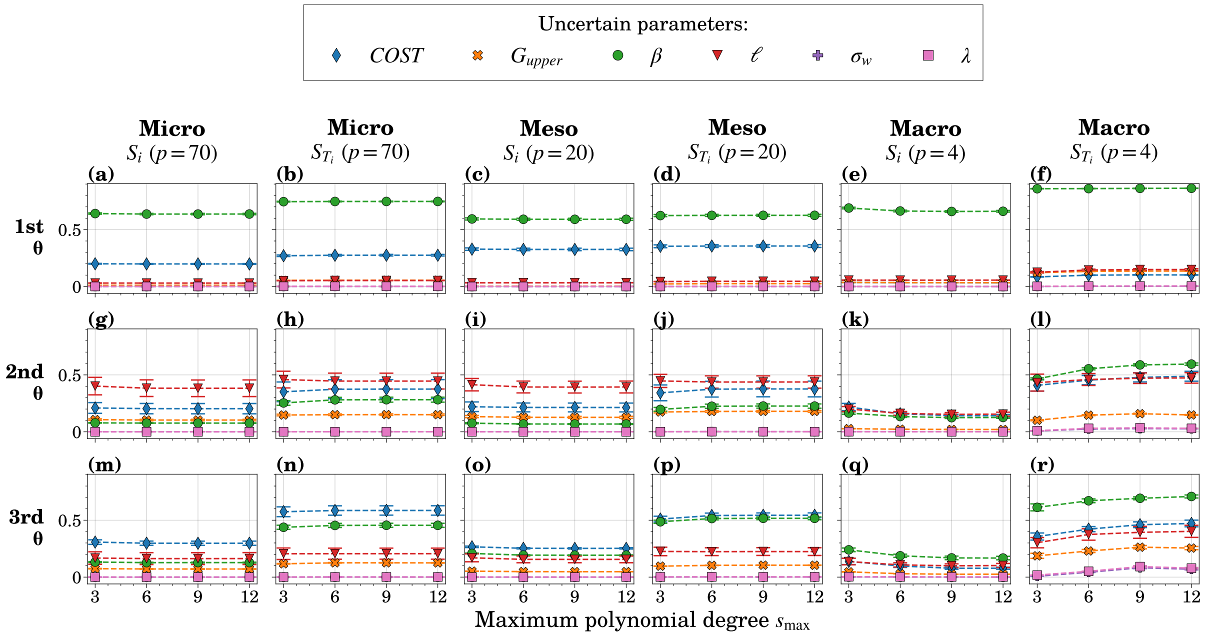

Supplementary Figure 21 Poverty trap formation ABM, micro, meso, and macro levels: estimates of first- and total-order sensitivity indices (S_i and S_{T_i}) of six uncertain input parameters (see Table 3 in Methods section of the main text) for four maximum polynomial degrees, $s_{\max} \in \{3, 6, 9, 12\}$. Sobol' indices were computed from PCE coefficients estimated with LAR on the manifold discovered with GDMaps using two Grassmannian dimensions of $p = 70$ for the micro level, $p = 20$ for the meso level, and $p = 4$ for the macro level. For the selection of diffusion coordinates, parsimonious representation was used. Error bars indicate variance across 10 runs.

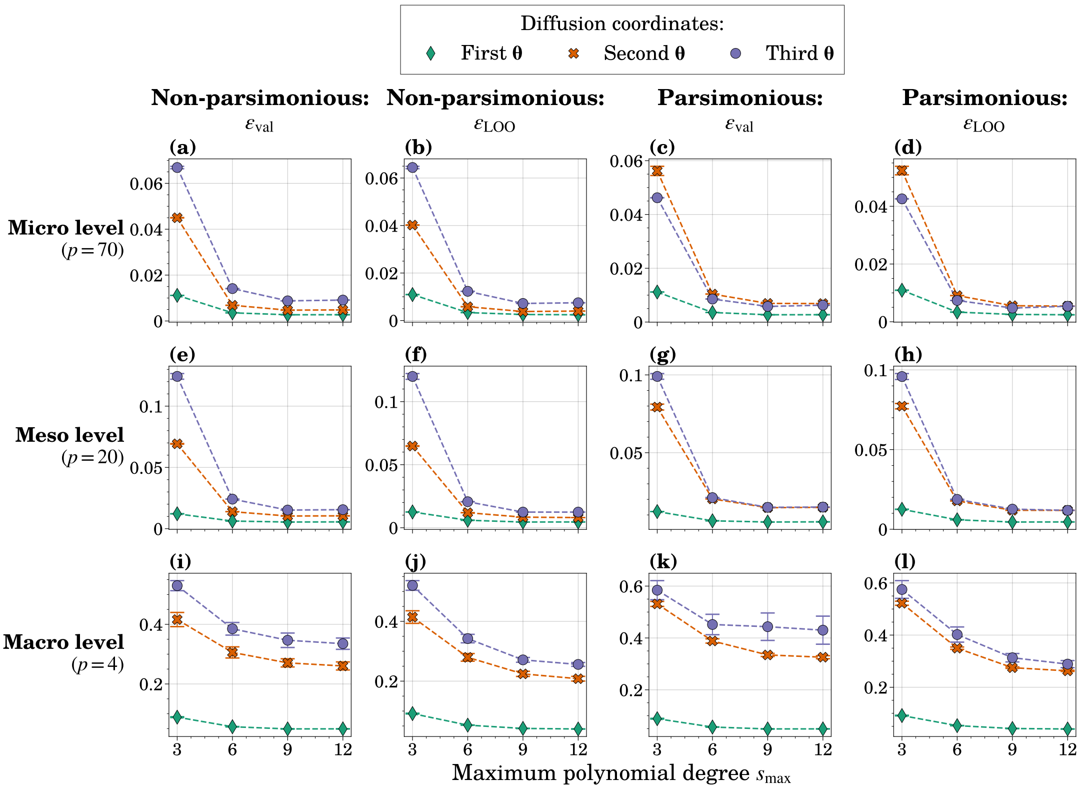

Supplementary Figure 22 Poverty trap formation ABM at the micro, meso and macro levels: validation and LOO errors (ϵ_{LOO} and ϵ_{val}, respectively) from GDMaps PCE using LAR for the three non-parsimoniously and parsimoniously selected diffusion coordinates and four maximum polynomial degrees, $s_{\max} \in \{3, 6, 9, 12\}$.

References

[1] Smith, R. C. *Uncertainty quantification: theory, implementation, and applications* Vol. 12 (Siam, 2013).

[2] Saltelli, A., Tarantola, S., Campolongo, F., Ratto, M. *et al. Sensitivity analysis in practice: a guide to assessing scientific models* Vol. 1 (Wiley Online Library, 2004).

[3] Thiele, J. C., Kurth, W. & Grimm, V. Facilitating parameter estimation and sensitivity analysis of agent-based models: A cookbook using netlogo and r. *Journal of Artificial Societies and Social Simulation* **17**, 11 (2014).

[4] Ten Broeke, G., Van Voorn, G. & Ligtenberg, A. Which sensitivity analysis method should i use for my agent-based model? *Journal of Artificial Societies and Social Simulation* **19**, 5 (2016).

[5] Schouten, M., Verwaart, T. & Heijman, W. Comparing two sensitivity analysis approaches for two scenarios with a spatially explicit rural agent-based model. *Environmental Modelling Software* **54**, 196–210 (2014).

[6] North, M. J. & Macal, C. M. *Managing business complexity: discovering strategic solutions with agent-based modeling and simulation* (Oxford University Press, 2007).

[7] Fonoberova, M., Fonoberov, V. A. & Mezić, I. Global sensitivity/uncertainty analysis for agent-based models. *Reliability Engineering & System Safety* **118**, 8–17 (2013).

[8] Borgonovo, E., Pangallo, M., Rivkin, J., Rizzo, L. & Siggelkow, N. Sensitivity analysis of agent-based models: a new protocol. *Computational and Mathematical Organization Theory* **28**, 52–94 (2022).

[9] Ligmann-Zielinska, A. *et al.* 'one size does not fit all': a roadmap of purpose-driven mixed-method pathways for sensitivity analysis of agent-based models. *Journal of Artificial Societies and Social Simulation* **23** (2020).

[10] Sudret, B. Global sensitivity analysis using polynomial chaos expansions. *Reliability engineering & system safety* **93**, 964–979 (2008).

[11] Saltelli, A. (ed.) *Global sensitivity analysis: the primer* (John Wiley, Chichester, England ; Hoboken, NJ, 2008). OCLC: ocn180852094.

[12] Ligmann-Zielinska, A., Kramer, D. B., Spence Cheruvelil, K. & Soranno, P. A. Using uncertainty and sensitivity analyses in socioecological agent-based models to improve their analytical performance and policy relevance. *PloS one* **9**, e109779 (2014).

[13] Pianosi, F. & Wagener, T. Distribution-based sensitivity analysis from a generic input-output sample. *Environmental Modelling & Software* **108**, 197–207 (2018).

[14] Pianosi, F. & Wagener, T. A simple and efficient method for global sensitivity analysis based on cumulative distribution functions. *Environmental Modelling Software* **67**, 1–11 (2015).

[15] Brown, D. G., Page, S., Riolo, R., Zellner, M. & Rand, W. Path dependence and the validation of agent-based spatial models of land use. *International journal of geographical information science* **19**, 153–174 (2005).

[16] Manson, S. M. Challenges in evaluating models of geographic complexity. *Environment and Planning B: Planning and Design* **34**, 245–260 (2007).

[17] Richiardi, M. G., Leombruni, R., Saam, N. J. & Sonnessa, M. A common protocol for agent-based social simulation. *Journal of artificial societies and social simulation* **9** (2006).

[18] Ligmann-Zielinska, A. & Sun, L. Applying time-dependent variance-based global sensitivity analysis to represent the dynamics of an agent-based model of land use change. *International Journal of Geographical Information Science* **24**, 1829–1850 (2010).

[19] Bone, C., Johnson, B., Nielsen-Pincus, M., Sproles, E. & Bolte, J. A temporal variant-invariant validation approach for agent-based models of landscape dynamics. *Transactions in GIS* **18**, 161–182 (2014).

[20] Campbell, K., McKay, M. D. & Williams, B. J. Sensitivity analysis when model outputs are functions. *Reliability Engineering & System Safety* **91**, 1468–1472 (2006).

[21] Lamboni, M., Monod, H. & Makowski, D. Multivariate sensitivity analysis to measure global contribution of input factors in dynamic models. *Reliability engineering & system safety* **96**, 450–459 (2011).

[22] Gamboa, F., Janon, A., Klein, T. & Lagnoux, A. Sensitivity analysis for multidimensional and functional outputs (2014).

[23] Garcia-Cabrejo, O. & Valocchi, A. Global sensitivity analysis for multivariate output using polynomial chaos expansion. *Reliability Engineering & System Safety* **126**, 25–36 (2014).

[24] Alexanderian, A., Gremaud, P. A. & Smith, R. C. Variance-based sensitivity analysis for time-dependent processes. *Reliability Engineering & System Safety* **196**, 106722 (2020).

[25] Nagel, J. B., Rieckermann, J. & Sudret, B. Principal component analysis and sparse polynomial chaos expansions for global sensitivity analysis and model calibration: Application to urban drainage simulation. *Reliability Engineering & System Safety* **195**, 106737 (2020).

[26] Li, M., Wang, R.-Q. & Jia, G. Efficient dimension reduction and surrogate-based sensitivity analysis for expensive models with high-dimensional outputs. *Reliability Engineering & System Safety* **195**, 106725 (2020).

[27] Perrin, T. *et al.* Functional principal component analysis for global sensitivity analysis of model with spatial output. *Reliability Engineering & System Safety* **211**, 107522 (2021).

[28] Jung, W. & Taflanidis, A. A. Efficient global sensitivity analysis for high-dimensional outputs combining data-driven probability models and dimensionality reduction. *Reliability Engineering & System Safety* **231**, 108805 (2023).

[29] Dos Santos, K. R., Giovanis, D. G. & Shields, M. D. Grassmannian diffusion maps–based dimension reduction and classification for high-dimensional data. *SIAM Journal on Scientific Computing* **44**, B250–B274 (2022).

[30] Kontolati, K., Loukrezis, D., dos Santos, K. R., Giovanis, D. G. & Shields, M. D. Manifold learning-based polynomial chaos expansions for high-dimensional surrogate models. *International Journal for Uncertainty Quantification* **12** (2022).

[31] Ligmann-Zielinska, A. Spatially-explicit sensitivity analysis of an agent-based model of land use change. *International Journal of Geographical Information Science* **27**, 1764–1781 (2013).

[32] McGibbon, R. T. & Pande, V. S. Variational cross-validation of slow dynamical modes in molecular kinetics. *The Journal of chemical physics* **142** (2015).

[33] Schütte, C., Fischer, A., Huisinga, W. & Deuflhard, P. A direct approach to conformational dynamics based on hybrid monte carlo. *Journal of Computational Physics* **151**, 146–168 (1999).

[34] Noé, F. & Nuske, F. A variational approach to modeling slow processes in stochastic dynamical systems. *Multiscale Modeling & Simulation* **11**, 635–655 (2013).

[35] Noé, F. & Rosta, E. Markov models of molecular kinetics. *The Journal of chemical physics* **151** (2019).

[36] Kim, S. B., Dsilva, C. J., Kevrekidis, I. G. & Debenedetti, P. G. Systematic characterization of protein folding pathways using diffusion maps: Application to trp-cage miniprotein. *The Journal of chemical physics* **142** (2015).

[37] Das, P., Moll, M., Stamati, H., Kavraki, L. E. & Clementi, C. Low-dimensional, free-energy landscapes of protein-folding reactions by nonlinear dimensionality reduction. *Proceedings of the National Academy of Sciences* **103**, 9885–9890 (2006).

[38] Ferguson, A. L., Panagiotopoulos, A. Z., Debenedetti, P. G. & Kevrekidis, I. G. Systematic determination of order parameters for chain dynamics using diffusion maps. *Proceedings of the National Academy of Sciences* **107**, 13597–13602 (2010).

[39] Coifman, R. R. *et al.* Geometric diffusions as a tool for harmonic analysis and structure definition of data: Diffusion maps. *Proceedings of the national academy of sciences* **102**, 7426–7431 (2005).

[40] Coifman, R. R. & Lafon, S. Diffusion maps. *Applied and computational harmonic analysis* **21**, 5–30 (2006).

[41] Zheng, W., Rohrdanz, M. A. & Clementi, C. Rapid exploration of configuration space with diffusion-map-directed molecular dynamics. *The journal of physical chemistry B* **117**, 12769–12776 (2013).

[42] Koopman, B. O. Hamiltonian systems and transformation in hilbert space. *Proceedings of the National Academy of Sciences* **17**, 315–318 (1931).

[43] Schmid, P. J. Dynamic mode decomposition of numerical and experimental data. *Journal of fluid mechanics* **656**, 5–28 (2010).

[44] Brunton, S. L., Brunton, B. W., Proctor, J. L. & Kutz, J. N. Koopman invariant subspaces and finite linear representations of nonlinear dynamical systems for control. *PloS one* **11**, e0150171 (2016).

[45] Kamb, M., Kaiser, E., Brunton, S. L. & Kutz, J. N. Time-delay observables for koopman: Theory and applications. *SIAM Journal on Applied Dynamical Systems* **19**, 886–917 (2020).

[46] Snyder, G. & Song, Z. Koopman operator theory for nonlinear dynamic modeling using dynamic mode decomposition. *arXiv preprint arXiv:2110.08442* (2021).

[47] Rowley, C. W., Mezić, I., Bagheri, S., Schlatter, P. & Henningson, D. S. Spectral analysis of nonlinear flows. *Journal of fluid mechanics* **641**, 115–127 (2009).
